# Misrepresentation of Neuroscience Data Might Give Rise to Misleading Conclusions in the Media: The Case of Attention Deficit Hyperactivity Disorder

**DOI:** 10.1371/journal.pone.0014618

**Published:** 2011-01-31

**Authors:** Francois Gonon, Erwan Bezard, Thomas Boraud

**Affiliations:** 1 Institut des Maladies Neurodégénératives, University of Bordeaux, Bordeaux, France; 2 Centre National de la Recherche Scientifique UMR 5293, Bordeaux, France; University of Ioannina School of Medicine, Greece

## Abstract

**Background:**

There is often a huge gap between neurobiological facts and firm conclusions stated by the media. Data misrepresentation in the conclusions and summaries of neuroscience articles might contribute to this gap.

**Methodology/Principal Findings:**

Using the case of attention deficit hyperactivity disorder (ADHD), we identified three types of misrepresentation. The first relies on prominent inconsistencies between results and claimed conclusions and was observed in two scientific reports dealing with ADHD. Only one out of the 61 media articles echoing both scientific reports adequately described the results and, thus questioned the claimed conclusion. The second type of misrepresentation consists in putting a firm conclusion in the summary while raw data that strongly limit the claim are only given in the results section. To quantify this misrepresentation we analyzed the summaries of all articles asserting that polymorphisms of the gene coding for the D4 dopaminergic receptor are associated with ADHD. Only 25 summaries out of 159 also mentioned that this association confers a small risk. This misrepresentation is also observed in most media articles reporting on ADHD and the D4 gene. The third misrepresentation consists in extrapolating basic and pre-clinical findings to new therapeutic prospects in inappropriate ways. Indeed, analysis of all ADHD-related studies in mice showed that 23% of the conclusions were overstated. The frequency of this overstatement was positively related with the impact factor of the journal.

**Conclusion/Significance:**

Data misrepresentations are frequent in the scientific literature dealing with ADHD and may contribute to the appearance of misleading conclusions in the media. In synergy with citation distortions and publication biases they influence social representations and bias the scientific evidence in favor of the view that ADHD is primarily caused by biological factors. We discuss the social consequences and the causes of data misrepresentations and suggest a few corrective actions.

## Introduction

“The problems often start with how research findings are presented to the public” said Prof G. McKhann [1]. Indeed, all neuroscientists have in mind “flashy stories” where media have presented weak or controversial findings as established conclusions. As in other fields of biomedical research journalists [2] and press releases [3] certainly contribute to this misrepresentation. Moreover, citation distortions and publications biases, which have been described in biomedical research [4]–[7] including neuroscience [8], also contribute to create unfounded authority of claims. Here, we point out the misrepresentation of the neurobiological facts at its initial level, i.e. inside individual scientific articles. Indeed, a fair and constructive debate requires that “authors are obligated to present their data in a form that minimizes the chance that readers will be misled about what was actually observed.” (Guide of the Society For Neuroscience: “Responsible Conduct Regarding Scientific Communication”, paragraph 1.13.2). We show how and to what extent this ethical commitment is not fulfilled in many neuroscience articles. As stated by Prof McKhann, data misrepresentation is an ethical concern for the neuroscience community: “If our advances are repeatedly overstated or over-promoted and public distrust of neuroscience grows, then we have only ourselves to blame.”

ADHD is considered to be the most common neuropsychiatric disorder of childhood with a prevalence rate of approximately 7–9%. Psychostimulants effectively alleviate symptoms in most ADHD children. Hundreds of studies have investigated the neurobiology of ADHD and numerous hypotheses have been proposed. The dopamine deficit theory is still the most popular one [9] although it has been questioned by others [10] and in our recent review article [11]. In the present study we do not question the data regarding ADHD and the validity of their interpretation. We examine how data are presented in scientific and media articles.

Whilst preparing our review on ADHD we noticed several types and cases of data misrepresentation. Therefore, we will mainly defend our view using the example of ADHD. However, there is no reason to think that data misrepresentation occurs only, or is worst, in this particular field. We identified three types of misrepresentation in the scientific literature about ADHD. The first relies on prominent inconsistencies between results and conclusions claimed in the title and summary. The second consists in putting a firm conclusion in the summary while raw data that strongly limit the claim are only given in the results section. In the third, basic and pre-clinical findings are extrapolated to new therapeutic prospects in inappropriate ways. Here, we illustrate each type of misrepresentation by analyzing scientific and media articles reporting on specific topics related to ADHD. Then, we discuss the social consequences and the causes of these misrepresentations. Finally, we suggest a few remedies.

## Results

### Internal inconsistencies

In our review of the ADHD literature [11], we have read about 360 articles and we have found only two studies showing obvious discrepancies between results and claimed conclusions [12], [13]. These internal inconsistencies have already been discussed in detail [11] and are summarized in Table 1. Our observation that only two articles among 360 show obvious internal inconsistencies must be considered with caution however. First, our review of the ADHD literature was not a systematic one and was not aimed at pointing out internal inconsistencies. Second, generalization to other fields of the neuroscience literature would be unjustified. We can only say that our observations confirm our intuition: this first type of misrepresentation is, fortunately, infrequent.

**Table 1:** In two articles showing internal inconsistencies only the claimed conclusion is echoed in the media.

**Article** title	Depressed dopamine activity in caudate and preliminary evidence of limbic involvement in adults with attention-deficit/hyperactivity disorder. *(Volkow et al., 2007)*	Modifiers of long-term school outcomes for children with attention-deficit/hyperactivity disorder: does treatment with stimulant medication make a difference? *(Barbaresi et al., 2007)*
Claim in the article	“These results provide evidence of depressed dopamine activity in ADHD.”… “The findings of reduced dopamine release in subjects with ADHD are consistent with the notion that the ability of stimulant medications to enhance extracellular dopamine underlies their therapeutic effects in ADHD.”	“This study supports the hypothesis that treatment with stimulant medication is associated with more favorable, long-term school outcomes for children with ADHD.”
Facts questioning the claim (article citations)	“D2/D3 receptor availability was significantly lower in subjects with ADHD…Since measures of D2/D3 availability are influenced by extracellular dopamine, low Bmax could reflect either increased dopamine release or low D2/D3 receptor levels.” “We cannot rule out the possibility that the blunted dopamine response to methylphenidate in subjects with ADHD could reflect higher baseline dopamine tone.”	“The average reading score at the time of the last assessment was similar between the groups of cases that were treated versus not treated with stimulant.” “The proportion of school dropout was similar between treated and not treated cases.”
**Newspapers** Title	“Brain chemicals have key role in ADHD, studies show”. *(The Wall Street Journal, August 7, 2007)*	“ADHD drugs help boost children's grades” *(Washington Post, September 21, 2007)*
citation	“A team led by Dr. Nora Volkow, director of the NIH's National Institute on Drug Abuse, documented decreased dopamine activity in the brains of a group of adults with ADHD. Volkow said the decreased dopamine activity related to systems involved with attention and cognition, but also with reward.”	“This is the first study that shows that taking stimulants for ADHD improves long-term school performance,” said lead researcher Dr. William Barbaresi.”
**Medical website:** title	ADHD appears to be associated with depressed dopamine activity in the brain. *(* http://www.docguide.com *)*	ADHD stimulant drug therapy helps improve long-term school outcomes. *(* http://www.news-medical.net *)*
citation	“The findings of reduced dopamine release in subjects with ADHD are consistent with the notion that the ability of stimulant medications to enhance extracellular dopamine underlies their therapeutic effects in ADHD,” the authors write.	“In this study, treatment with stimulant medication during childhood was associated with more favorable long-term school outcomes,” explains William Barbaresi,

The point of interest here is that both articles have been echoed in the media as shown in Table 1. The media almost always reported on the claimed conclusion. Indeed, concerning the article by Volkow et al (2007), we have checked 40 media articles and the conclusion that dopamine is depressed in the brain of ADHD patients has been always reported. We have never read a mitigating statement saying that their results are open to the opposite interpretation although the authors explicitly raised this possibility in their result section (Table 1). In our sample of 21 articles that reported on the study by Barbaresi et al (2007) in the media, only one (*The Guardian*, London, September 21, 2007) adequately described the results and, thus questioned the conclusion claimed by Barbaresi's group (Table 1).

More surprisingly, the scientific literature is no more critical. Between its publication and February 2010 the study by Volkow et al (2007) has been cited 30 times in scientific articles. Among them, 20 articles cited the conclusion that dopamine activity is depressed in ADHD without further comment. Apart from our review article [11], none of them pointed out its internal inconsistency.

### Fact omission

This misrepresentation consists of putting in the summary a fixed conclusion while raw data, which strongly limit the relevance of this conclusion, are only given in the result section. To quantify this misrepresentation, we have extensively studied how the scientific literature reports on a specific issue: the association between alleles of the gene coding for the D4 dopamine receptor (DRD4) and ADHD.

To fully appraise this misrepresentation it is illuminating to compare a statement in the media and the corresponding facts. The health guide of the *New York Times* says: “Genetic factors may play the most important role in ADHD…. Most of the research on the underlying genetic mechanisms targets the neurotransmitter dopamine. Variations in genes that regulate specific dopamine receptors have been identified *in a high proportion of people* with ADHD.” Actually, “the most robust finding in ADHD is the association of a variable number tandem repeat polymorphism in exon 3 of the DRD4 gene” [14]. However, although the 7-repeat allele is significantly associated with ADHD, it confers small risk [15]: ADHD patients have a higher frequency of this allele as compared to controls, 23% versus 17%, respectively [16]. Therefore, there is a huge gap between the media statement and the neurobiological facts. This gap is generated when scientific texts report the association of the 7-repeat allele of the DRD4 gene with ADHD but do not mention at the same time that it confers small risk.

To quantify this misrepresentation in the scientific literature, we examined the summaries of all 219 articles about ADHD that mentioned the DRD4 gene. Articles were classified between review articles (52), animal (or in vitro) studies (24) and research articles in humans (143). This third category was further divided into articles, in which genetic data related to the DRD4 were provided (117) or not (26). In this second category, as well as in animal studies, statements related to the association of the DRD4 gene with ADHD thus corresponded to citations of other articles. In these 219 summaries we counted the presence of specific statements as indicated in Table 2.

**Table 2:** Content analysis of the summaries of scientific articles containing “ADHD” and “D4” or “DRD4”.

Statements	Review articles	Animal studies	Human studies*	
			citation	data
Total number of articles	52	24	26	117
1) DRD4 gene is associated with ADHD	37	17	25	55
2) DRD4 gene is associated but it confers small risk	6	0	0	19
3) DRD4 is not associated with ADHD	1	1	0	27
4) Not relevant	8	6	1	16
Ratio (small risk)/(DRD4 associated) i.e. (1/1+2)	6/43	0/17	0/25	19/74
Omission rate: (1-ratio) ×100	86%	100%	100%	74%

*Human studies were divided into articles providing data on the DRD4 gene (“data”) or not (“citation”).

The presence of the following statements was numbered.

*1) “DRD4 associated with ADHD”*. In these articles, the association of the DRD4 gene with ADHD is stated as an established fact.

*2) “DRD4 gene is associated but it confers small risk”.* In these articles the first statement is mitigated by either mentioning raw data (e.g. odds ratio) or by stating that the DRD4 gene confers small risk to ADHD.

*3) “DRD4 is not associated with ADHD”.* These articles defend the view that the association of ADHD with the DRD4 gene does not reach statistical significance.

*4) “Not relevant”.* In these articles the summary was not informative enough to know whether the authors defend the view that the DRD4 gene is associated with ADHD.

Among the 117 primary studies in humans, 74 articles state in their summary that alleles of the DRD4 genes are significantly associated with ADHD but only 19 summaries also mentioned that they confer a small risk. One may argue that summaries are too short to report the details. However, almost the same number of summaries (14) did not mention that it confers small risk but reinforced the view that genetic factors play the most important role in ADHD with an additional statement about its high heritability. Moreover, this misrepresentation always occurs in the summaries of primary articles that cite the association of the DRD4 gene with ADHD but do not report data on it (Table 2).

This misrepresentation is even more robust in review articles. Among the 43 relevant summaries stating that the DRD4 gene is significantly associated with ADHD only 6 mentioned that the 7-repeat allele confers a small risk. Again one may argue that this is due to length constraints, but this explanation is not consistent with other observations. Indeed, 13 summaries did not mention that it confers a small risk but added a statement on the high heritability of ADHD. Likewise, 9 summaries also mentioned the following type of erroneous statement: “The efficacy of stimulant agents confirms that the neurotransmitter abnormalities seen in ADHD are primarily catecholaminergic in origin.” The weakness of this argument has long been underlined [11], [17], [18] and relies on the fact that psychostimulants enhance attention to the same extent both in ADHD and healthy children [17].

On the whole, the case of the association between ADHD and the DRD4 gene shows that the omission of relevant facts limiting the impact of the claim is not restricted to a few scientific articles: it occurs in a vast majority of the summaries. Although in most reports and review articles, the raw data (e.g. odds ratios) were given inside the results section, it is likely that many readers may not check inside the text the relevance of the statement put in the summary (“the DRD4 gene is associated with ADHD”).

This misrepresentation is also observed in media articles. Indeed, we looked for press articles reporting on the DRD4 gene and on ADHD. Among 170 relevant articles published from 1996 to 2009, all but 2 stated that polymorphisms of the DRD4 gene are significantly associated with ADHD. Twenty-five articles also mentioned either the raw data or that it confers small risk, while 117 articles did not. Furthermore, 26 articles mentioned the odds ratio (from 1.2 to 1.34) but also put an overstated conclusion (e.g. “These findings strongly implicate the involvement of brain dopamine systems in the pathogenesis of ADHD.”). Thus, the 26 equivocal articles being discarded, 82% of the media articles misrepresented the association between the DRD4 gene and ADHD. This omission rate is very similar to that observed in scientific articles (Table 2).

The literature on the association between the DRD4 gene and ADHD further exemplifies a major publication bias: the most robust effects are reported in initial studies [5]. Indeed, although this association is still considered to be highly statistically significant, its odds ratio decreased with successive studies from 2.4 in the oldest study in 1996 [19] to reach 1.27 in the most recent meta-analysis [15]. This decrease in the clinical relevance of this association is not correlated with parallel changes in type-2 misrepresentation. Indeed, omission rates both in scientific and media articles did not decrease over the years 1996 to 2009 (Table S1).

### Extrapolating basic and pre-clinical findings to new therapeutic prospects

**Figure 1:**
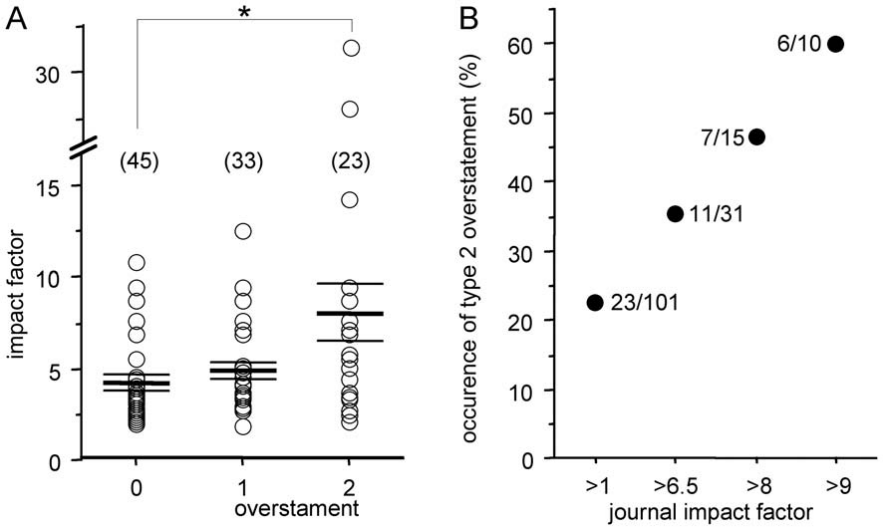
Overstatement of the relevance of mouse studies towards ADHD neurobiology and treatment. Studies were selected with a systematic search via PubMed (see methods). We rated a study as overstated when the link between ADHD and the studied mice only relied on their behavioral similarities with ADHD symptoms and when the conclusion stated that the findings provide novel insights into the neurobiology of ADHD. When this overstatement was reinforced by a claim about the clinical relevance of the study, it was rated as of type 2. Among the 101 studies examined, 56 were classified as overstated (33 type 1 and 23 type 2). **A**. Relationship between these 3 classes and the impact factor of the corresponding journal. Horizontal bars indicate mean ± SEM for the 3 classes. This impact factor was significantly higher (ANOVA, F = 6.52, Fisher's test: *p = 0.0006) when comparing studies with the type 2 overstatement to studies without overstatement. **B**. The occurrence rate of extrapolating to new therapeutic prospects (type 2 overstatement) is positively related to the impact factor.

This third type of misrepresentation is illustrated with three examples concerning ADHD (Table 3). Unjustified overstatements are frequent in the conclusions of studies with animal models. As an example, we examined a survey of all ADHD-related studies reporting data from the mouse brain. We rated a study as overstated when the link between ADHD and the studied mice only relied on their behavioral similarities with ADHD symptoms and when the conclusion stated that the findings provide novel insights into the neurobiology of ADHD (see Methods). Indeed, because ADHD is a very complex disease associated in most patients with other psychiatric disorders (e.g. anxiety, depression, conduct disorders), investigations based on mouse behavior cannot capture the ADHD complexity. From our survey of 101 articles we found that only 45 were not overstated and that 23 studies also extrapolate to new therapeutic prospects. These 23 overstated studies were published in journals with a significantly higher impact factor (Fig. 1A–B). When they are published in high rank journals these overstatements are often echoed in the media as exemplified in Table 3. We examined 63 media articles that echoed the 3 articles mentioned in Table 3. We observed that they faithfully reported these 3 overstatements although a few (11/63) also added a comment that mitigated it (see two examples in Table 3).

**Table 3:** Examples of extrapolating basic findings to new therapeutic prospects and their echoes in the media.

**Science**: Title	Dopamine transporter density in patients with attention deficit hyperactivity disorder.	Role of serotonin in the paradoxical calming effect of psychostimulants on hyperactivity.	Impulsive choice induced in rats by lesions of the nucleus accumbens core.
*Ref.*	*Lancet (1999)* [26]	*Science (1999)* [27]	*Science (2001)* [28]
over-statement	“The dopamine transporter in brain, a major target of the majority of drugs used to treat ADHD, was elevated by about 70% compared to healthy controls. The use of ^129^I altropane SPECT could be expanded to individualize treatment.”	“The preponderance of common symptomatologies between DAT-KO mice and individuals with ADHD suggests that these mice may not only serve as a useful animal model and as a resource to test new therapies but that they may also provide insights into the basic mechanisms that underlie the etiology of this and other hyperkinetic disorders.”	“Impulsive choice contributes to drug addiction, ADHD… Thus, dysfunction of the nucleus accumbens core may be a key element in the neuropathology of impulsivity.”
comment	This study was based on only 6 adults with ADHD. Whether the DAT level is altered in ADHD patients is still a matter of debate (see [29]).	DAT-KO mice are calmed by psychostimulants via the inhibition of the serotonin transporter. However, specific inhibitors of the serotonin transporter do not alleviate ADHD symptoms [30], [31].	A recent meta-analysis “do not support simpler models which posit that ADHD is strictly a disorder resulting from deficits of activity in a few isolated brain regions” [32].
**Media**: Title	Brain scans seen as test in attention disorder	Findings: Better attention deficit drugs possible	Pleasing find on gratification.
*source*	*The Boston Globe*	*The Washington Post*	*Financial Times*
date	December 17, 1999	January 15, 1999	May 25, 2001
citation	Brain scans have identified a clear-cut chemical abnormality in people with ADHD, …It could be a first step toward a long-sought test for attention- deficit hyperactivity disorder, say researchers.	It may be possible to design better drugs for treating ADHD, which affects millions of children in the United States, researchers said yesterday. Tests on mice show that stimulant drugs currently used to treat the disorder, such as Ritalin and amphetamines, work in a more complex way than previously thought, the researchers said.	The discovery could help research into drug addiction, attention-deficit disorder, hyperactivity and other personality disorders that are marked by inability to control instant gratification.
Mitigating citation	“This is certainly not yet a diagnostic test,” because it involved a “very refined sample” of patients who aren't representative of the entire spectrum of those who have the disorder, Barkley said.	The researchers did not, however, measure serotonin levels in the mice. And mice are physically very different from humans and often react differently to drugs.	
*Number of articles	35 (including 9 mitigating comments)	20 (2 mitigating comments)	8 (no mitigating comment)

*In this last row we give the number of media articles obtained using a systematic search (see Methods) that echoed the corresponding scientific articles. In parentheses we indicate the number of media articles that added a mitigating statement.

## Discussion

### Limitations of the study

Using the case of ADHD, we investigated 3 types of data misrepresentation in scientific articles: internal inconsistencies, omission of relevant facts and unjustified extrapolation to new therapeutic prospects. The first type was illustrated with only two scientific reports and this selection does not result from a systematic search. Therefore, our observations cannot give a quantitative estimate of the prevalence of this misrepresentation. The second and the third types of misrepresentation were each illustrated with only one specific aspect of the ADHD literature. In both cases we analyzed a corpus of scientific reports selected by a systematic search. Therefore, our study is mainly qualitative and does not provide quantitative information about the extent of data misrepresentation in the ADHD literature as a whole.

Our examples of data misrepresentation in scientific reports seem to be correlated with similar misrepresentation in the lay media. Thus, we speculate that data misrepresentation in the scientific literature might play a part in the distortion of data into misleading conclusions in the media. In support of our hypothesis, we observed that many lay articles either cite the conclusions stated in scientific articles or report interviews of the scientific authors.

### Data misrepresentation, citation distortion and publication bias

Data misrepresentations in the summaries and conclusions seem to spread in media articles. Indeed, we noticed only a few discrepancies between the conclusions stated in scientific articles and how they are echoed in the media. Overstatements to therapeutic prospects are faithfully reported although some reservations are sometimes expressed. As previously suspected [20], we show here with one example that making an overstatement to therapeutic prospects is positively correlated with publication in leading journals. In the biomedical literature, scientific articles, which are echoed in the media, are more likely to be cited in subsequent scientific articles [21]. These circular relationships may explain why these overstatements are advantageous both for authors and editors.

Citation distortion has been already studied in detail regarding a specific neurobiological claim [8]. We also observed several case of citation distortion in the ADHD literature. For example, the facts that psychostimulants both alleviate symptoms in most ADHD children and enhance the extracellular dopamine level have repeatedly been put forward to support the dopamine deficit theory of ADHD. However, this inference is rarely mitigated by also mentioning that psychostimulants induce the same behavioral effects in healthy children [17].

We also observed in the ADHD literature several examples of a major publication bias: the most robust effects are reported in initial studies. This publication bias has already been pointed out in the biomedical literature [4], [5] and is obvious regarding the association of ADHD either with the DRD4 gene or with the density of the dopamine transporter. Here we show that data misrepresentation reinforces this publication bias in both scientific and media articles. Indeed, whereas subsequent studies diminish the odds ratio and thus, the clinical relevance of the initial study, the rate of type-2 misrepresentation (omitting to mention relevant data) does not decrease with time.

Although most journal guidelines explicitly condemn them, data misrepresentations, citation distortions and publication biases are frequent in the neuroscience literature. They “provide a distorted view of the reality of scientific data” and reinforce “dominant themes [that] lead to stagnating conformism” [7]. Regarding the neurobiology of ADHD, although histamine neurons do play a role in attention, it is striking to note that the number of studies on dopamine (1314) dramatically exceeds those on histamine (38) (PubMed March 2010, key words: ADHD, dopamine, histamine). Review articles are expected to provide a wider and more balanced view of a theme. Unfortunately, most review articles tend to confine themselves to consensual points of view [8]. In conclusion, we believe that neuroscientists contribute to distorting data into misleading conclusions in the media. However, most neuroscientists do not seem to be conscious that these distortions have social consequences.

### Social and public health consequences

There is no doubt that ADHD is a real concern in the sense that most ADHD diagnosed individuals suffer from attention deficit and excessive impulsivity. According to Singh (2008) there are three partially overlapping positions in the public debate about ADHD. The first one posits that ADHD is primarily caused by biological factors, the second that ADHD is caused by a combination of biological and social factors and the third that ADHD is primarily caused by environmental factors [22]. The first position is not consistent with data demonstrating that environmental factors play a role in ADHD (low economic status, severe child mistreatment, maternal smoking during pregnancy, premature birth, teenager pregnancy and other environmental adversities) [22].

Unfortunately, data misrepresentation biases the scientific evidence in favor of the first position stating that ADHD is primarily caused by biological factors. Therefore, this misrepresentation does have social consequences regarding ADHD management: it favors medical interventions over prevention and psycho-social interventions. Moreover, the first position favors research programs that seek to identify biomarkers associated with an ADHD risk. Although no biomarker has yet been already validated in psychiatry, “the current interest in biomarkers is a sign that psychiatry has undergone a methodological shift, away from searching for the causes of a condition towards estimating the probability that the condition is present or will develop” [23]. According to this view, “children might be subject to intrusive medical interventions that focus on individual-level risk factors rather than on social and environmental risk factors” [23]. Likewise, the neuroscience discourse about depression biases the public debate in favor of biological causes and promotes the use of antidepressants [18], [24]. This excessive support of the medicalization of psychic suffering induces counter-reactions promoting alternative responses such as psychotherapies, but also often leads to suspicious attitudes towards neuroscience and might promote irrational beliefs and conducts in society.

The social consequences also depend on the type of misrepresentation. The first type (internal inconsistencies) and the second (omission of significant facts) do have large consequences when they are echoed in the media. Indeed, they corrupt the message received by the general public, including medical doctors. The third type (exaggerated extrapolations to therapeutic prospects) seems at first glance the least damaging. However, when it occurs in animal studies, it implicitly supports the view that drug treatments are the only solution to mental disorders. Indeed, animal models are not suitable to develop psycho-social interventions. Moreover, and probably far more damaging in the long term to neuroscience, this misrepresentation feeds illusory short-term hopes in patients and their families. For example, animal studies about cellular therapies for spinal cord injury have been put forward by for-profit institutions selling these therapies to unfortunate patients although “these interventions are not yet proven safe and effective by properly conducted clinical trials” [25]. Extrapolation of basic findings to therapeutic prospects never acknowledges the lag time of 10 to 15 years needed for making a novel treatment available to the public, even should the concept be further validated in subsequent studies. Examples of such overstatements are far too abundant to be listed and basically apply to all neurological and psychiatric conditions.

### Causes of data misrepresentation

Most neuroscientists believe that extrapolating their basic finding to new therapeutic prospects will help their study to be published in a high impact factor journal. Accordingly, our study of the case of ADHD suggests that this type of overstatement is positively associated with publication in prestigious journals. Although it is difficult to know whether this association is causal, there is no neuroscience journal, to our knowledge, stating in their instructions to authors that extrapolation to therapeutic prospects is acceptable only if fully justified. More generally, competition between authors to publish in high rank journals and between journal editors distorts publication of biomedical research in favor of sensationalism [7]. Pressures to publish in leading journals have been already discussed and include careerism and evaluation of science by bibliographic indicators [20].

Moreover, neuroscience is closely linked to related medical sciences, neurology, psychiatry and neuropharmacology. National research agencies increasingly restrict their support to research projects with potential applications in these 3 medical domains. Therefore, neuroscientists are encouraged to work in line with this institutional demand and to preferentially publish positive results. Even when they are published, negative results or data challenging established dogma are often ignored both by the scientific literature and by the media as exemplified here. Data misrepresentation, citation distortion and publication biases feed the information cascades, which are circularly used to justify the hypotheses put forward in grant proposals [8]. “Once research funding has been used to join a cascade there are further incentives to interpret results through confirmation bias to demonstrate success of the research for subsequent funding” [8].

### Solutions

First, because our pioneer study is mainly qualitative, we need more studies analyzing the neuroscience discourse. These studies must receive sufficient visibility to draw the attention of the neuroscience community to the extent of data misrepresentation and to its negative consequences. As neuroscience findings are increasingly echoed by the media, we are now, and much more so than in the past, in the public eye. Distortions of neuroscience findings open the door to suspicious public attitudes towards neuroscience and this might result in a decrease of the resources that society will accept being allocated to future research. It is the responsibility of the neuroscience community, and in its long-term interest, to correct this as soon as possible. Second, the key regulators of our publication system are the journal editors. If they collectively reject sensationalism and clearly condemn data misrepresentation, we may expect rapid improvement. This might involve changes in current publication practices as suggested [7], [20]. Third, the neuroscience community should lobby in favor of research grants without any link to therapeutic applications. This lobbying should explain to politicians that an excessive support of therapeutically oriented research programs is counterproductive because it favors herding research and encourages misinformation of the lay public.

## Methods

### Sources of data

Our analysis was restricted to scientific articles published in English in journal issues whose publication year was 2009 or earlier. Articles without abstracts were not considered. They were collected from PubMed by means of systematic searches, except for the two articles analyzed in the first section *“Internal inconsistencies”*. Both articles were found on the occasion of a previous review article about the neurobiology of ADHD. Because this previous work was not a systematic review and because our aim was not to carefully look for all articles exhibiting internal inconsistencies between their results sections and conclusions, both articles represent examples of internal inconsistencies. In other words our study does not provide a reliable quantitative estimate of this particular type of data misrepresentation. In the same section we used the site “ISI Web of Knowledge” to find the 30 scientific articles citing the study by Volkow et al (2007). Because only 8 studies cited the article by Barbaresi et al (2007), we did not analyze them.

In the third section of the results entitled “*Extrapolating basic and pre-clinical findings to new therapeutic prospects*” the impact factor of scientific journals was provided by “ISI Web of Knowledge” using the tool “Journal Citation Reports”. We used the impact factors given by this tool for 2008.

Articles published in English in newspapers and magazines were systematically searched using the web site “Dow Jones Factiva” (http://global.factiva.com/sb/default.aspx?NAPC=S&fcpil=fr) and appropriate key words, whose presence was searched in the full texts. Primary scientific articles also given by this database were discarded. When very similar articles were published in distinct newspapers or magazines they were considered as distinct articles. Articles published on web sites did not originate from a systematic search and were found using “Google” and appropriate key words.

### Data selection and classification

Articles published in newspapers, magazines or web sites and reporting either on the study by Volkow et al (2007) or on that by Barbaresi et al (2007) (Table 1) were found using the following key words: either Volkow or Barbaresi AND (hyperactivity OR ADHD). The search periods were restricted to articles published in August, September and October 2007. The corresponding reference lists are given in Text S1. We examined these 61 articles to determine whether they only reported on the claimed conclusion.

We examined the summaries of scientific articles that mentioned ADHD and the DRD4 gene. Articles were selected via PubMed using the following equation applied to all fields: (attention deficit hyperactivity disorder OR ADHD) AND (D4 OR DRD4). From the 235 retrieved articles we discarded 16 articles whose abstracts did not mentioned DRD4. The remaining 219 summaries were classified as described in Table 2. The corresponding reference lists are given in Text S2, according to that classification.

Articles published in newspapers and magazines from January 1996 to January 2010 were selected using the following criteria: hyperactivity AND (D4 OR DRD4). Articles, which were not informative enough to know whether the authors have defended the view that the DRD4 gene is associated with ADHD were discarded. The 170 remaining articles were examined and classified according to the same rules as described in Table 2. The classified lists of references are given in Text S2.

All articles related to ADHD and reporting on experiments involving the mouse brain were selected via PubMed with the following search equation applied to all fields using the PubMed limit *“animals”*: (attention deficit hyperactivity disorder OR ADHD) AND (mouse OR mice). This search retrieved 178 articles published in 2009 or earlier. We discarded 58 review articles, 16 studies that reported observations not related to the mouse brain and 3 studies not related to ADHD. In the 101 selected articles we examined the conclusions stated in the summary and at the end of the discussion. We rated a study as overstated when the link between ADHD and the studied mice only relied on their behavioral similarities with ADHD symptoms and when the conclusion stated that the findings have provided novel insights into the neurobiology of ADHD. When this overstatement was reinforced by a claim about the clinical relevance of the study, it was rated as of type 2. Studies, which were considered as not overstated, belonged to 3 types: i) mice submitted to experimental conditions mimicking those causally involved in ADHD (e.g. lead toxicity, maternal deprivation), ii) investigations into the effects of psychostimulant treatments on the mouse brain and iii) studies on mouse models of ADHD, in which authors refrained from overstating their conclusion. This classification was double-checked independently by two investigators. The classified lists of references are given in Text S3.

Using the web site “Dow Jones Factiva” we looked for media articles explicitly reporting on the three scientific studies mentioned in Table 3 by Dougherty et al (1999), Gainetdinov et al (1999) and Cardinal et al (2001). The search criteria were (hyperactivity OR ADHD) combined either with (scans OR scan), with (mice) or with (Cardinal OR rats), respectively. The search periods were 3 month long and started one day before the date of online publication of the corresponding scientific article. Using this procedure we have collected 63 media articles explicitly echoing to the 3 corresponding scientific articles (Table 3). After collection of these 63 media articles, each article was compared to the overstatement expressed in the corresponding scientific article. The classified lists of references are given in Text S3.

## Supporting Information

Table S1 Distribution of the omission rate with the publication year.
(0.04 MB DOC)

Text S1 Lists of media and scientific articles echoing the studies by Volkow et al (2007) and Barbaresi et al (2007) shown in Table 1.
(0.08 MB DOC)

Text S2 Scientific and media articles reporting on the association between alleles of the gene coding for the D4 dopamine receptor and ADHD.
(0.14 MB DOC)

Text S3 Scientific studies performed in mice and related to ADHD and media articles that echoed to the 3 scientific articles given in Table 3.
(0.08 MB DOC)

## References

[1] McKhann G (2007). Research Must Pass an Ethical ‘Smell Test’.. http://www.dana.org/news/braininthenews/detail.aspx?id=9928.

[2] Dentzer S (2009). Communicating medical news—pitfalls of health care journalism.. N Engl J Med.

[3] Woloshin S, Schwartz LM (2002). Press releases: translating research into news.. Jama.

[4] Easterbrook PJ, Berlin JA, Gopalan R, Matthews DR (1991). Publication bias in clinical research.. Lancet.

[5] Ioannidis JP (2005). Contradicted and initially stronger effects in highly cited clinical research.. Jama.

[6] Dwan K, Altman DG, Arnaiz JA, Bloom J, Chan AW (2008). Systematic review of the empirical evidence of study publication bias and outcome reporting bias.. PLoS One.

[7] Young NS, Ioannidis JP, Al-Ubaydli O (2008). Why current publication practices may distort science.. PloS Medicine.

[8] Greenberg SA (2009). How citation distortions create unfounded authority: analysis of a citation network.. BMJ 339:.

[9] Swanson JM, Kinsbourne M, Nigg J, Lanphear B, Stefanatos GA (2007). Etiologic subtypes of attention-deficit/hyperactivity disorder: brain imaging, molecular genetic and environmental factors and the dopamine hypothesis.. Neuropsychology Review.

[10] Pliszka SR, McCracken JT, Maas JW (1996). Catecholamines in attention-deficit hyperactivity disorder: current perspectives.. J Am Acad Child Adolesc Psychiatry.

[11] Gonon F (2009). The dopaminergic hypothesis of attention-deficit/hyperactivity disorder needs re-examining.. Trends in Neuroscience.

[12] Barbaresi WJ, Katusic SK, Colligan RC, Weaver AL, Jacobsen SJ (2007). Modifiers of long-term school outcomes for children with attention-deficit/hyperactivity disorder: does treatment with stimulant medication make a difference? Results from a population-based study.. J Dev Behav Pediatr.

[13] Volkow ND, Wang GJ, Newcorn J, Telang F, Solanto MV (2007a). Depressed dopamine activity in caudate and preliminary evidence of limbic involvement in adults with attention-deficit/hyperactivity disorder.. Archives of General Psychiatry.

[14] Heiser P, Friedel S, Dempfle A, Konrad K, Smidt J (2004). Molecular genetic aspects of attention-deficit/hyperactivity disorder.. Neuroscience and Biobehavioral Reviews.

[15] Gizer IR, Ficks C, Waldman ID (2009). Candidate gene studies of ADHD: a meta-analytic review.. Hum Genet.

[16] Gornick MC, Addington A, Shaw P, Bobb AJ, Sharp W (2007). Association of the dopamine receptor D4 (DRD4) gene 7-repeat allele with children with attention-deficit/hyperactivity disorder (ADHD): an update.. Am J Med Genet B Neuropsychiatr Genet.

[17] Rapoport JL, Buchsbaum MS, Zahn TP, Weingartner H, Ludlow C (1978). Dextroamphetamine: cognitive and behavioral effects in normal prepubertal boys.. Science.

[18] Valenstein ES (1988). Blaming the brain..

[19] LaHoste GJ, Swanson JM, Wigal SB, Glabe C, Wigal T (1996). Dopamine D4 receptor gene polymorphism is associated with attention deficit hyperactivity disorder.. Mol Psychiatry.

[20] Lawrence PA (2003). The politics of publication.. Nature.

[21] Phillips DP, Kanter EJ, Bednarczyk B, Tastad PL (1991). Importance of the lay press in the transmission of medical knowledge to the scientific community.. N Engl J Med.

[22] Singh I (2008). Beyond polemics: science and ethics of ADHD.. Nature Review Neuroscience.

[23] Singh I, Rose N (2009). Biomarkers in psychiatry.. Nature.

[24] Horwitz AV, Wakefield JC (2007). The loss of sadness: how psychiatry transformed normal sorrow into depressive disorder..

[25] Blight A, Curt A, Ditunno JF, Dobkin B, Ellaway P (2009). Position statement on the sale of unproven cellular therapies for spinal cord injury: the international campaign for cures of spinal cord injury paralysis.. Spinal Cord.

[26] Dougherty DD, Bonab AA, Spencer TJ, Rauch SL, Madras BK (1999). Dopamine transporter density in patients with attention deficit hyperactivity disorder.. Lancet.

[27] Gainetdinov RR, Wetsel WC, Jones SR, Levin ED, Jaber M (1999). Role of serotonin in the paradoxical calming effect of psychostimulants on hyperactivity.. Science.

[28] Cardinal RN, Pennicott DR, Sugathapala CL, Robbins TW, Everitt BJ (2001). Impulsive choice induced in rats by lesions of the nucleus accumbens core.. Science.

[29] Volkow ND, Wang GJ, Newcorn J, Fowler JS, Telang F (2007b). Brain dopamine transporter levels in treatment and drug naive adults with ADHD.. Neuroimage.

[30] Findling RL (1996). Open-label treatment of comorbid depression and attentional disorders with co-administration of serotonin reuptake inhibitors and psychostimulants in children, adolescents, and adults: a case series.. Journal of American Academy of Child & Adolescent Psychiatry.

[31] Popper CW (1997). Antidepressants in the treatment of attention-deficit/hyperactivity disorder.. Journal of Clinical Psychiatry.

[32] Dickstein SG, Bannon K, Castellanos FX, Milham MP (2006). The neural correlates of attention deficit hyperactivity disorder: an ALE meta-analysis.. J Child Psychol Psychiatry.

