# Supplementary material for: Misrepresentation of Neuroscience Data Might Give Rise to Misleading Conclusions in the Media: The Case of Attention Deficit Hyperactivity Disorder
Source: PLoS One. 2011 Jan 31;6(1):e14618. doi: 10.1371/journal.pone.0014618 (PMC3031509; doi:10.1371/journal.pone.0014618)
Supplement: Table S1 — Distribution of the omission rate with the publication year. (0.04 MB DOC) [file pone.0014618.s001.doc]

**Table S1.** Distribution of the omission rate with the publication year.

|  | Total number of article | | omission rate (%) | |
| --- | --- | --- | --- | --- |
|  | Scientific | media | Scientific | media |
| 1996-1997 | 2 | 10 | 100 | 20 |
| 1998-1999 | 9 | 4 | 78 | 75 |
| 2000-2001 | 22 | 4 | 73 | 75 |
| 2002-2003 | 15 | 11 | 87 | 91 |
| 2004-2005 | 38 | 17 | 87 | 100 |
| 2006-2007 | 35 | 61 | 86 | 80 |
| 2008-2009 | 38 | 34 | 89 | 97 |

In this table we only considered scientific summaries and full media articles stating that the DRD4 gene is associated with ADHD. As in table 2 we distinguished articles, which have put this statement without further comment or data (fact omission) from those, in which it has been mitigated by either mentioning raw data (e.g. odds ratio) or by stating that the DRD4 gene confers small risk to ADHD. From these data we calculated the omission rate, i.e. the percentage of articles, which omitted to put a mitigating comment. The table shows the distribution of these data with the publication year. The omission rate does not decrease from 1996 to 2009 both in scientific summaries and in media articles.
