# Supplementary material for: Misrepresentation of Neuroscience Data Might Give Rise to Misleading Conclusions in the Media: The Case of Attention Deficit Hyperactivity Disorder
Source: PLoS One. 2011 Jan 31;6(1):e14618. doi: 10.1371/journal.pone.0014618 (PMC3031509; doi:10.1371/journal.pone.0014618)
Supplement: Text S1 — Lists of media and scientific articles echoing the studies by Volkow et al (2007) and Barbaresi et al (2007) shown in Table 1. (0.08 MB DOC) [file pone.0014618.s002.doc]

**Supporting Text S1**

**1) Lists of media articles echoing the study by Volkow et al (2007a)**

*1.1) Articles in newspapers and magazines*

Attention Deficit Hyperactivity Disorder Therapy; Reports from National Institute on Drug Abuse advance knowledge in attention deficit hyperactivity disorder therapy. September 8, 2007. *Obesity, Fitness & Wellness Week*

Adults with attention-deficit/hyperactivity disorder appear to be associated with depressed dopamine activity in brain. August 6, 2007. *US Fed News*

Adult ADHD linked to decreased dopamine activity in the brain. August 7, 2007. *Asian News International*

Brain chemical tied to ADHD in two studies. August 7, 2007. *The Wall Street Journal*

Reduced dopamine activity seen in brains of adults with condition. August 8, 2007. *American Health Line*

ADHD appears to be associated with depressed dopamine activity in the brain. August 20, 2007. *Pharma Business Week*

ADHD appears to be associated with depressed dopamine activity in the brain. August 20, 2007. *Mental Health Weekly Digest*

ADHD appears to be associated with depressed dopamine activity in the brain. August 20, 2007. *Health & Medicine Week*

ADHD appears to be associated with depressed dopamine activity in the brain. August 20, 2007. *Biotech Business Week*

ADHD appears to be associated with depressed dopamine activity in the brain. August 21, 2007. *Science Letter*

Attention Deficit Hyperactivity Disorder; ADHD Appears to Be Associated With Depressed Dopamine Activity in the Brain. August 21, 2007. *Life Science Weekly*

Attention Deficit Hyperactivity Disorder; ADHD Appears to Be Associated With Depressed Dopamine Activity in the Brain. August 22, 2007. *Biotech Week*

Attention Deficit Hyperactivity Disorder; ADHD Appears to Be Associated With Depressed Dopamine Activity in the Brain. August 24, 2007. *Drug Week*

Attention Deficit Hyperactivity Disorder; ADHD Appears to Be Associated With Depressed Dopamine Activity in the Brain. August 25, 2007. *Obesity, Fitness & Wellness Week*

Attention Deficit Hyperactivity Disorder Therapy; Reports from National Institute on Drug Abuse advance knowledge in attention deficit hyperactivity disorder therapy. September 7, 2007. *Drug Week*

Attention Deficit Hyperactivity Disorder Therapy; Reports from National Institute on Drug Abuse advance knowledge in attention deficit hyperactivity disorder therapy. September 3, 2007. *Pharma Business Week*

Attention Deficit Hyperactivity Disorder Therapy; Reports from National Institute on Drug Abuse advance knowledge in attention deficit hyperactivity disorder therapy. September 3, 2007. *Health & Medicine Week*

Attention Deficit Hyperactivity Disorder Therapy; Reports from National Institute on Drug Abuse advance knowledge in attention deficit hyperactivity disorder therapy. September 3, 2007. *Mental Health Weekly Digest*

Attention Deficit Hyperactivity Disorder Therapy; Reports from National Institute on Drug Abuse advance knowledge in attention deficit hyperactivity disorder therapy. September 3, 2007. *Biotech Business Week*

Attention Deficit Hyperactivity Disorder Therapy; Reports from National Institute on Drug Abuse advance knowledge in attention deficit hyperactivity disorder therapy. September 4, 2007. *Science Letter*

Attention Deficit Hyperactivity Disorder Therapy; Reports from National Institute on Drug Abuse advance knowledge in attention deficit hyperactivity disorder therapy. September 4, 2007. *Life Science Weekly*

Attention Deficit Hyperactivity Disorder Therapy; Reports from National Institute on Drug Abuse advance knowledge in attention deficit hyperactivity disorder therapy. September 5, 2007. *Biotech Week*

Brain chemical has key role in ADHD, studies show. August 6, 2007. *Reuters News*

PET scans show depressed dopamine activity in ADHD. October 1, 2007. *Clinical Psychiatry News*

*1.2) Articles in web sites*

A new study has linked ADHD in adults with depressed dopamine activity in the brain. *http://www.medindia.net/news/Decreased-Dopamine-Activity-in-Brain-Leads-to-ADHD-in-Adults-24668-2.htm*

ADHD seems to be linked to low dopamine brain activity. *http://www.medicalnewstoday.com/articles/79070.php*

ADHD appears to be associated with depressed dopamine activity in the brain. *http://news.bio-medicine.org/medicine-news-3/ADHD-appears-to-be-associated-with-depressed-dopamine-activity-in-the-brain-113-2/*

Depressed dopamine activity in caudate and preliminary evidence of limbic involvement in adults with ADHD. August, 2007. *http://newideas.net/adhd_blog/%5Btag%5D/summer_studies_2007_1*

ADHD is "Real". August, 2007. *http://www.yalemedicalgroup.org/news/2_807.html*

Brain studies show ADHD is real disease: condition appears linked to dopamine function, experts say. August 6, 2007. *http://health.usnews.com/usnews/health/healthday/070806/brain-studies-show-adhd-is-real-disease.htm*

Two new studies show that a brain chemical called dopamine may play a role in attention deficit hyperactivity disorder (ADHD). August 6, 2007. *http://www.webmd.com/add-adhd/news/20070806/new-clues-on-causes-of-adhd*

ADHD appears to be associated with depressed dopamine activity in the brain. August 6, 2007. *http://www.docguide.com/news/content.nsf/news/852571020057CCF68525732F0049D025*

ADHD appears to be associated with depressed dopamine activity in the brain. August 6, 2007. *http://www.bnl.gov/bnlweb/pubaf/pr/PR_display.asp?prID=07-X12*

Dopamine influences ADHD. August 7, 2007. *http://psychcentral.com/news/2007/08/07/dopamine-influences-adhd/1107.html*

Dopamine has key role in ADHD. August 7, 2007. *http://archive.newsmax.com/archives/articles/2007/8/7/85936.shtml*

ADHD appears to be associated with depressed dopamine activity in the brain. August 8, 2007. *http://www.sciencedaily.com/releases/2007/08/070806164505.htm*

Brain chemical has key role in ADHD. August 9, 2007. *http://www.pchrd.dost.gov.ph/library/index.php/news-archive/525*

In a recent study, compared with healthy controls, adults with attention-deficit/hyperactivity disorder (ADHD) had less dopamine released in the caudate. August 9, 2007. *http://www.medscape.com/viewarticle/561202*

ADHD linked to dopamine levels. August 13, 2007. *http://www.additudemag.com/addnews/42/2696.html*

PET scans show depressed dopamine activity in ADHD. October, 2007. *http://findarticles.com/p/articles/mi_hb4345/is_10_35/ai_n29385330/*

**2) Lists of media articles echoing the study by Barbaresi et al (2007)**

*2.1) Articles in newspapers and magazines*

ADHD medication linked to improved academic results in kids. September 18, 2007. *Asian News International*

Mayo Clinic study indicates medication for AD/HD may help student outcomes. September 18, 2007. *PR Newswire (U.S.)*

Drug treatment helps children with ADHD keep up at school. September 21, 2007. *The Guardian*

ADHD drugs help boost children's grades. September 21, 2007. *The Washington Post*

ADHD; Reports from College of Medicine describe recent advances in attention deficit hyperactivity disorder in children. October 1, *Science Letter*

ADHD; Reports from College of Medicine describe recent advances in attention deficit hyperactivity disorder in children. October 2, 2007. *Life Science Weekly*

ADHD; Reports from College of Medicine describe recent advances in attention deficit hyperactivity disorder in children. October 1, 2007. *Health & Medicine Week*

ADHD; Reports from College of Medicine describe recent advances in attention deficit hyperactivity disorder in children. October 3, 2007. *Biotech Week*

ADHD; Reports from College of Medicine describe recent advances in attention deficit hyperactivity disorder in children. October 6, 2007. *Obesity, Fitness & Wellness Week*

2.2) Articles in web sites

ADHD drugs help boost children's grades. *http://pluk.mt.typepad.com/pluk_news_feed/medication/*

Mayo Clinic: ADHD medication associated with improved academic performance. September 19, 2007 *http://www.foxnews.com/story/0,2933,297298,00.html*

Drug treatment improves long-term school success, study shows. September 19, 2007 *http://www.medicinenet.com/script/main/art.asp?articlekey=84032*

Medication for ADHD may help student outcomes. September 27, 2007 *https://www.achievesolutions.net/achievesolutions/en/ctbhp/Content.do?contentId=11209*

Mayo Clinic study indicates medication for AD/HD may help student outcomes. September 19, 2007. *http://www.medicalnewstoday.com/articles/82892.php*

ADHD meds may improve academics. September 19, 2007. *http://psychcentral.com/news/2007/09/19/adhd-meds-may-improve-academics/1304.html*

ADHD stimulant drug therapy helps improve long-term school outcomes. September 19, 2007. *http://www.news-medical.net/?id=30047*

ADHD drugs help boost children's grades. September 21, 2007. *http://health.usnews.com/usnews/health/healthday/070921/adhd-drugs-help-boost-childrens-grades.htm*

ADHD drugs help boost children's grades. September 21, 2007. *http://sexualhealth.ehealthsource.com/index.php?p=news1&id=608450*

ADHD drugs help boost children's grades. September 21, 2007. *http://www.bio-medicine.org/medicine-news-1/ADHD-Drugs-Help-Boost-Childrens-Grades-1741-2/*

Medication for ADHD may help students succeed at school. September 24, 2007. *http://www.sciencedaily.com/releases/2007/09/070918144259.htm*

ADHD medication linked to improved academic results in kids. September 19, 2007. *http://news.webindia123.com/news/ar_showdetails.asp?id=709190654&cat=&n_date=20070919*

**3) Scientifc articles citing the study by Volkow et al (2007a)**

*3.1) Articles citing, without further comment, the conclusion that dopamine is depressed in ADHD*

Antai-Otong, D (2008) Pharmacological management of adult ADHD: Implications for psychiatric care. *Perspectives In Psychiatric Care* 44: 196-201.

Arnsten, AFT (2009a) The Emerging Neurobiology of Attention Deficit Hyperactivity Disorder: The Key Role of the Prefrontal Association Cortex. *Journal Of Pediatrics* 154: S22-S31.

Arnsten, AFT (2009b) Toward a New Understanding of Attention-Deficit Hyperactivity Disorder Pathophysiology An Important Role for Prefrontal Cortex Dysfunction. *Cns Drugs* 23: 33-41.

Findling, RL, Arnold, LE, Greenhill, LL, Kratochvil, CJ, McGough, JJ (2007) Diagnosing and managing complicated ADHD. *Journal Of Clinical Psychiatry* 68: 1963-1970.

Gilden, DL, Marusich, LR (2009) Contraction of Time in Attention-Deficit Hyperactivity Disorder. *Neuropsychology* 23: 265-269.

Herrmann, MJ *et al* (2009) Emotional deficits in adult ADHD patients: an ERP study. *Soc Cogn Affect Neurosci* 4: 340-345.

Ludolph, AG *et al* (2008) Dopaminergic dysfunction in attention deficit hyperactivity disorder (ADHD), differences between pharmacologically treated and never treated young adults: A 3,4-dihdroxy-6-[F-18] fluorophenyl-L-alanine PET study. *Neuroimage* 41: 718-727.

Lule, D, Ludolph, AC, Ludolph, AG (2008) Neurodevelopmental and neurodegenerative diseases - Is there a pathophysiological link? Attention-deficit/hyperactivity disorder and amyotrophic lateral sclerosis as examples. *Medical Hypotheses* 70: 1133-1138.

Nikolaus, S, Antke, C, Muller, HW (2009) In vivo imaging of synaptic function in the central nervous system: II. Mental and affective disorders. *Behavioural Brain Research* 204: 32-66.

Oades, RD *et al* (2008) The influence of serotonin- and other genes on impulsive behavioral aggression and cognitive impulsivity in children with attention-deficit/hyperactivity disorder (ADHD): Findings from a family-based association test (FBAT) analysis. *Behavioral And Brain Functions* 4.

Oner, P, Dirik, EB, Taner, Y, Caykoylu, A, Anlar, O (2007) Association between low serum ferritin and restless legs syndrome in patients with attention deficit hyperactivity disorder. *Tohoku Journal Of Experimental Medicine* 213: 269-276.

Oner, P, Oner, O (2008) Relationship of ferritin to symptom ratings children with Attention Deficit Hyperactivity Disorder: Effect of comorbidity. *Child Psychiatry & Human Development* 39: 323-330.

Pinkhardt, EH *et al* (2009) Intensified testing for attention-deficit hyperactivity disorder (ADHD) in girls should reduce depression and smoking in adult females and the prevalence of ADHD in the longterm. *Medical Hypotheses* 72: 409-412.

Prince, J (2008) Catecholamine dysfunction in attention-deficit/hyperactivity disorder - An update. *Journal Of Clinical Psychopharmacology* 28: S39-S45.

Schott, BH *et al* (2008) Mesolimbic Functional Magnetic Resonance Imaging Activations during Reward Anticipation Correlate with Reward-Related Ventral Striatal Dopamine Release. *Journal Of Neuroscience* 28: 14311-14319.

Vaidya, CJ, Stollstorff, M (2008) Cognitive Neuroscience Of Attention Deficit Hyperactivity Disorder: Current Status And Working Hypotheses. *Developmental Disabilities Research Reviews* 14: 261-267.

Volkow, ND, Fowler, JS, Wang, GJ, Baler, R, Telang, F (2009a) Imaging dopamine's role in drug abuse and addiction. *Neuropharmacology* 56: 3-8.

Volkow, ND, Swanson, JM (2008) Does childhood treatment of ADHD with stimulant medication affect substance abuse in adulthood? *American Journal Of Psychiatry* 165: 553-555.

Volkow, ND *et al* (2009b) Evaluating Dopamine Reward Pathway in ADHD Clinical Implications. *Jama-Journal Of The American Medical Association* 302: 1084-1091.

Volz, TJ, Farnsworth, SJ, Rowley, SD, Hanson, GR, Fleckenstein, AE (2008) Methylphenidate-induced increases in vesicular dopamine sequestration and dopamine release in the striatum: The role of muscarinic and dopamine D2 receptors. *Journal Of Pharmacology And Experimental Therapeutics* 327: 161-167.

*3.2) Articles citing the study by Volkow et al (2007a) but without any statement about a dopamine deficit in ADHD patients.*

Brotman, MA *et al* (2010) Amygdala activation during emotion processing of neutral faces in children with severe mood dysregulation versus ADHD or bipolar disorder. *Am J Psychiatry* 167: 61-69.

Covey, L, Manubay, J, Jiang, HP, Nortick, M, Palumbo, D (2008) Smoking cessation and inattention or hyperactivity/impulsivity: A post hoc analysis. *Nicotine & Tobacco Research* 10: 1717-1725.

Egerton, A *et al* (2009) The dopaminergic basis of human behaviors: A review of molecular imaging studies. *Neuroscience And Biobehavioral Reviews* 33: 1109-1132.

Ellison-Wright, I, Ellison-Wright, Z, Bullmore, E (2008) Structural brain change in Attention Deficit Hyperactivity Disorder identified by meta-analysis. *Bmc Psychiatry* 8.

Gruber, R *et al* (2009) Dopamine Transporter Genotype and Stimulant Side Effect Factors in Youth Diagnosed with Attention-Deficit/Hyperactivity Disorder. *Journal Of Child And Adolescent Psychopharmacology* 19: 233-239.

Plichta, MM *et al* (2009) Neural Hyporesponsiveness and Hyperresponsiveness During Immediate and Delayed Reward Processing in Adult Attention-Deficit/Hyperactivity Disorder. *Biological Psychiatry* 65: 7-14.

Schaefers, AT, Teuchert-Noodt, G, Bagorda, F, Brummelte, S (2009) Effect of postnatal methamphetamine trauma and adolescent methylphenidate treatment on adult hippocampal neurogenesis in gerbils. *European Journal Of Pharmacology* 616: 86-90.

Scherk, H *et al* (2008) SNAP-25 genotype influences NAA/Cho in left hippocampus. *Journal Of Neural Transmission* 115: 1513-1518.

Wilens, TE (2008) Effects of methylphenidate on the catecholaminergic system in attention-deficit/hyperactivity disorder. *Journal Of Clinical Psychopharmacology* 28: S46-S53.

*3.3) Article pointing out the internal inconsistency of the study by Volkow et al (2007a)*

Gonon, F (2009) The dopaminergic hypothesis of attention-deficit/hyperactivity disorder needs re-examining. *Trends In Neurosciences* **32**: 2-8.
