# Supplementary material for: Misrepresentation of Neuroscience Data Might Give Rise to Misleading Conclusions in the Media: The Case of Attention Deficit Hyperactivity Disorder
Source: PLoS One. 2011 Jan 31;6(1):e14618. doi: 10.1371/journal.pone.0014618 (PMC3031509; doi:10.1371/journal.pone.0014618)
Supplement: Text S2 — Scientific and media articles reporting on the association between alleles of the gene coding for the D4 dopamine receptor and ADHD. (0.14 MB DOC) [file pone.0014618.s003.doc]

**Supporting Text S2**

**A) Scientific articles reporting on the association between alleles of the gene coding for the D4 dopamine receptor and ADHD.**

**1) Review articles (52 articles)**

*1.1) Review articles with the first statement: "D4 gene is associated with ADHD"*

Asherson, P, Kuntsi, J, Taylor, E (2005) Unravelling the complexity of attention-deficit hyperactivity disorder: a behavioural genomic approach. *Br J Psychiatry* **187**: 103-105.

Asherson, PJ, Curran, S (2001) Approaches to gene mapping in complex disorders and their application in child psychiatry and psychology. *Br J Psychiatry* **179**: 122-128.

Bellgrove, MA, Mattingley, JB (2008) Molecular genetics of attention. *Ann N Y Acad Sci* **1129**: 200-212.

Biederman, J, Faraone, SV (2002) Current concepts on the neurobiology of Attention-Deficit/Hyperactivity Disorder. *J Atten Disord* **6 Suppl 1**: S7-16.

Biederman, J, Spencer, T (1999) Attention-deficit/hyperactivity disorder (ADHD) as a noradrenergic disorder. *Biol Psychiatry* **46**: 1234-1242.

Comings, DE (2001) Clinical and molecular genetics of ADHD and Tourette syndrome. Two related polygenic disorders. *Ann N Y Acad Sci* **931**: 50-83.

Curatolo, P, Paloscia, C, D'Agati, E, Moavero, R, Pasini, A (2009) The neurobiology of attention deficit/hyperactivity disorder. *Eur J Paediatr Neurol*. **13**: 299-304.

DiMaio, S, Grizenko, N, Joober, R (2003) Dopamine genes and attention-deficit hyperactivity disorder: a review. *J Psychiatry Neurosci* **28**: 27-38.

Durston, S, de Zeeuw, P, Staal, WG (2009) Imaging genetics in ADHD: A focus on cognitive control. *Neurosci Biobehav Rev*. **33**: 674-689.

Faraone, SV, Biederman, J (1998) Neurobiology of attention-deficit hyperactivity disorder. *Biol Psychiatry* **44**: 951-958.

Faraone, SV, Khan, SA (2006) Candidate gene studies of attention-deficit/hyperactivity disorder. *J Clin Psychiatry* **67 Suppl 8**: 13-20.

Faraone, SV *et al* (2005) Molecular genetics of attention-deficit/hyperactivity disorder. *Biol Psychiatry* **57**: 1313-1323.

Gizer, IR, Ficks, C, Waldman, ID (2009) Candidate gene studies of ADHD: a meta-analytic review. *Hum Genet* **126**: 51-90.

Hartman, DS, Lanau, F (1997) Diversity of dopamine receptors: new molecular and pharmacological developments. *Pol J Pharmacol* **49**: 191-199.

Heiser, P *et al* (2004) Molecular genetic aspects of attention-deficit/hyperactivity disorder. *Neurosci Biobehav Rev* **28**: 625-641.

Kebir, O, Tabbane, K, Sengupta, S, Joober, R (2009) Candidate genes and neuropsychological phenotypes in children with ADHD: review of association studies. *J Psychiatry Neurosci* **34**: 88-101.

Kent, L (2004) Recent advances in the genetics of attention deficit hyperactivity disorder. *Curr Psychiatry Rep* **6**: 143-148.

Kopeckova, M, Paclt, I, Goetz, P (2006) Polymorphisms and low plasma activity of dopamine-beta-hydroxylase in ADHD children. *Neuro Endocrinol Lett* **27**: 748-754.

Kuntsi, J, McLoughlin, G, Asherson, P (2006) Attention deficit hyperactivity disorder. *Neuromolecular Med* **8**: 461-484.

Levinson, DF (2005) Meta-analysis in psychiatric genetics. *Curr Psychiatry Rep* **7**: 143-151.

McGeary, J (2009) The DRD4 exon 3 VNTR polymorphism and addiction-related phenotypes: A review. *Pharmacol Biochem Behav*.

McGough, JJ (2005) Attention-deficit/hyperactivity disorder pharmacogenomics. *Biol Psychiatry* **57**: 1367-1373.

Mehler-Wex, C, Riederer, P, Gerlach, M (2006) Dopaminergic dysbalance in distinct basal ganglia neurocircuits: implications for the pathophysiology of Parkinson's disease, schizophrenia and attention deficit hyperactivity disorder. *Neurotox Res* **10**: 167-179.

Oades, RD (2008) Dopamine-serotonin interactions in attention-deficit hyperactivity disorder (ADHD). *Prog Brain Res* **172**: 543-565.

Oak, JN, Oldenhof, J, Van Tol, HH (2000) The dopamine D(4) receptor: one decade of research. *Eur J Pharmacol* **405**: 303-327.

Paclt, I *et al* (2005) Biochemical markers and genetic research of ADHD. *Neuro Endocrinol Lett* **26**: 423-430.

Prince, J (2008) Catecholamine dysfunction in attention-deficit/hyperactivity disorder: an update. *J Clin Psychopharmacol* **28**: S39-45.

Sharp, SI, McQuillin, A, Gurling, HM (2009) Genetics of attention-deficit hyperactivity disorder (ADHD). *Neuropharmacology* **57**: 590-600.

Swanson, J, Castellanos, FX, Murias, M, LaHoste, G, Kennedy, J (1998) Cognitive neuroscience of attention deficit hyperactivity disorder and hyperkinetic disorder. *Curr Opin Neurobiol* **8**: 263-271.

Swanson, JM *et al* (2000) Dopamine genes and ADHD. *Neurosci Biobehav Rev* **24**: 21-25.

Swanson, JM *et al* (2007) Etiologic subtypes of attention-deficit/hyperactivity disorder: brain imaging, molecular genetic and environmental factors and the dopamine hypothesis. *Neuropsychol Rev* **17**: 39-59.

Tarazi, FI, Baldessarini, RJ (1999) Dopamine D4 receptors: significance for molecular psychiatry at the millennium. *Mol Psychiatry* **4**: 529-538.

Tarazi, FI, Zhang, K, Baldessarini, RJ (2004) Dopamine D4 receptors: beyond schizophrenia. *J Recept Signal Transduct Res* **24**: 131-147.

Thapar, A, Holmes, J, Poulton, K, Harrington, R (1999) Genetic basis of attention deficit and hyperactivity. *Br J Psychiatry* **174**: 105-111.

Thapar, A, Langley, K, Owen, MJ, O'Donovan, MC (2007) Advances in genetic findings on attention deficit hyperactivity disorder. *Psychol Med* **37**: 1681-1692.

Thapar, A, O'Donovan, M, Owen, MJ (2005) The genetics of attention deficit hyperactivity disorder. *Hum Mol Genet* **14 Spec No. 2**: R275-282.

Thapar A, Stergiakouli E (2008) An overview on the genetics of ADHD. *Xin Li Xue Bao* **40**:1088- 1098.

*1.2) Review articles with the 2^nd^ statement: "D4 gene is associated but it confers small risk"*

Bobb, AJ, Castellanos, FX, Addington, AM, Rapoport, JL (2005) Molecular genetic studies of ADHD: 1991 to 2004. *Am J Med Genet B Neuropsychiatr Genet* **132B**: 109-125.

Coghill, D, Banaschewski, T (2009) The genetics of attention-deficit/hyperactivity disorder. *Expert Rev Neurother* **9**: 1547-1565.

Elia, J, Devoto, M (2007) ADHD genetics: 2007 update. *Curr Psychiatry Rep* **9**: 434-439.

Hawi, Z, Kirley, A, Lowe, N, Fitzgerald, M, Gill, M (2003) Recent genetic advances in ADHD and diagnostic and therapeutic prospects. *Expert Rev Neurother* **3**: 453-464.

Plomp, E, Van Engeland, H, Durston, S (2009) Understanding genes, environment and their interaction in attention-deficit hyperactivity disorder: is there a role for neuroimaging? *Neuroscience* **164**: 230-240.

Swanson, J *et al* (2001) Genes and attention deficit hyperactivity disorder. *Curr Psychiatry Rep* **3**: 92-100.

*1.3) Review articles with the 3^rd^ statement: " D4 is not associated with ADHD"*

Paterson, AD, Sunohara, GA, Kennedy, JL (1999) Dopamine D4 receptor gene: novelty or nonsense? *Neuropsychopharmacology* **21**: 3-16.

*1.4) Review articles not relevant to the present investigation*

Alsobrook, JP, 2nd, Pauls, DL (1998) Molecular approaches to child psychopathology. *Hum Biol* **70**: 413-432.

Faraone, SV (2003) Report from the 4th international meeting of the attention deficit hyperactivity disorder molecular genetics network. *Am J Med Genet B Neuropsychiatr Genet* **121B**: 55-59.

Ferguson, SS (2003) Receptor tyrosine kinase transactivation: fine-tuning synaptic transmission. *Trends Neurosci* **26**: 119-122.

Ficks, CA, Waldman, ID (2009) Gene-environment interactions in attention-deficit/hyperactivity disorder. *Curr Psychiatry Rep* **11**: 387-392.

Goldman, D, Lappalainen, J, Ozaki, N (1996) Direct analysis of candidate genes in impulsive behaviours. *Ciba Found Symp* **194**: 139-152; discussion 152-134.

Heijtz, RD, Kolb, B, Forssberg, H (2007) Motor inhibitory role of dopamine D1 receptors: implications for ADHD. *Physiol Behav* **92**: 155-160.

Levy, F (2002) Molecular genetics of ADHD: prospects for novel therapies. *Expert Rev Neurother* **2**: 491-497.

Viggiano, D, Ruocco, LA, Sadile, AG (2003) Dopamine phenotype and behaviour in animal models: in relation to attention deficit hyperactivity disorder. *Neurosci Biobehav Rev* **27**: 623-637.

**2) Animal studies (24 articles)**

*2.1) Articles with the 1^st^ statement: "D4 gene is associated with ADHD"*

Acosta-Garcia, J *et al* (2009) D4 and D1 dopamine receptors modulate [3H] GABA release in the substantia nigra pars reticulata of the rat. *Neuropharmacology* **57**: 725-730.

Avale, ME *et al* (2004) The dopamine D4 receptor is essential for hyperactivity and impaired behavioral inhibition in a mouse model of attention deficit/hyperactivity disorder. *Mol Psychiatry* **9**: 718-726.

Bergauer, M, Hubner, H, Gmeiner, P (2002) 2,4-Disubstituted pyrroles: synthesis, traceless linking and pharmacological investigations leading to the dopamine D4 receptor partial agonist FAUC 356. *Bioorg Med Chem Lett* **12**: 1937-1940.

Hejjas, K *et al* (2007) Association of polymorphisms in the dopamine D4 receptor gene and the activity-impulsivity endophenotype in dogs. *Anim Genet* **38**: 629-633.

James, AS *et al* (2007) Dimensions of impulsivity are associated with poor spatial working memory performance in monkeys. *J Neurosci* **27**: 14358-14364.

Li, Q *et al* (2007) The usefulness of the spontaneously hypertensive rat to model attention-deficit/hyperactivity disorder (ADHD) may be explained by the differential expression of dopamine-related genes in the brain. *Neurochem Int* **50**: 848-857.

Mill, J, Sagvolden, T, Asherson, P (2005) Sequence analysis of Drd2, Drd4, and Dat1 in SHR and WKY rat strains. *Behav Brain Funct* **1**: 24.

Nayak, S, Cassaday, HJ (2003) The novel dopamine D4 receptor agonist (PD 168,077 maleate): doses with different effects on locomotor activity are without effect in classical conditioning. *Prog Neuropsychopharmacol Biol Psychiatry* **27**: 441-449.

Noain, D *et al* (2006) Identification of brain neurons expressing the dopamine D4 receptor gene using BAC transgenic mice. *Eur J Neurosci* **24**: 2429-2438.

Powell, SB, Paulus, MP, Hartman, DS, Godel, T, Geyer, MA (2003) RO-10-5824 is a selective dopamine D4 receptor agonist that increases novel object exploration in C57 mice. *Neuropharmacology* **44**: 473-481.

Thomas, TC, Grandy, DK, Gerhardt, GA, Glaser, PE (2009) Decreased dopamine D4 receptor expression increases extracellular glutamate and alters its regulation in mouse striatum. *Neuropsychopharmacology* **34**: 436-445.

Van Craenenbroeck, K *et al* (2006) Influence of the antipsychotic drug pipamperone on the expression of the dopamine D4 receptor. *Life Sci* **80**: 74-81.

Woolley, ML *et al* (2008) Selective dopamine D4 receptor agonist (A-412997) improves cognitive performance and stimulates motor activity without influencing reward-related behaviour in rat. *Behav Pharmacol* **19**: 765-776.

Yuen, EY, Yan, Z (2009) Dopamine D4 receptors regulate AMPA receptor trafficking and glutamatergic transmission in GABAergic interneurons of prefrontal cortex. *J Neurosci* **29**: 550-562.

Zhang, K, Davids, E, Tarazi, FI, Baldessarini, RJ (2002a) Effects of dopamine D4 receptor-selective antagonists on motor hyperactivity in rats with neonatal 6-hydroxydopamine lesions. *Psychopharmacology (Berl)* **161**: 100-106.

Zhang, K, Tarazi, FI, Baldessarini, RJ (2001) Role of dopamine D(4) receptors in motor hyperactivity induced by neonatal 6-hydroxydopamine lesions in rats. *Neuropsychopharmacology* **25**: 624-632.

Zhang, K, Tarazi, FI, Davids, E, Baldessarini, RJ (2002b) Plasticity of dopamine D4 receptors in rat forebrain: temporal association with motor hyperactivity following neonatal 6-hydroxydopamine lesioning. *Neuropsychopharmacology* **26**: 625-633.

*2.2) No article with the second statement: "D4 gene is associated but it confers small risk"*

*2.3) Articles with the 3rd statement: "D4 is not associated with ADHD"*

Helms, CM, Gubner, NR, Wilhelm, CJ, Mitchell, SH, Grandy, DK (2008) D4 receptor deficiency in mice has limited effects on impulsivity and novelty seeking. *Pharmacol Biochem Behav* **90**: 387-393.

*2.4) Articles not relevant to the present investigation*

Browman, KE *et al* (2005) A-412997, a selective dopamine D4 agonist, improves cognitive performance in rats. *Pharmacol Biochem Behav* **82**: 148-155.

Li, Q *et al* (2009) Gene expression of synaptosomal-associated protein 25 (SNAP-25) in the prefrontal cortex of the spontaneously hypertensive rat (SHR). *Biochim Biophys Acta* **1792**: 766-776.

Masuo, Y, Ishido, M, Morita, M, Oka, S (2002) Effects of neonatal 6-hydroxydopamine lesion on the gene expression profile in young adult rats. *Neurosci Lett* **335**: 124-128.

Masuo, Y, Ishido, M, Morita, M, Oka, S (2004a) Effects of neonatal treatment with 6-hydroxydopamine and endocrine disruptors on motor activity and gene expression in rats. *Neural Plast* **11**: 59-76.

Masuo, Y, Morita, M, Oka, S, Ishido, M (2004b) Motor hyperactivity caused by a deficit in dopaminergic neurons and the effects of endocrine disruptors: a study inspired by the physiological roles of PACAP in the brain. *Regul Pept* **123**: 225-234.

Rhodes, JS, Garland, T (2003) Differential sensitivity to acute administration of Ritalin, apomorphine, SCH 23390, but not raclopride in mice selectively bred for hyperactive wheel-running behavior. *Psychopharmacology (Berl)* **167**: 242-250.

**3) Studies in humans that did not report data about the DRD4 gene (26)**

*3.1) Articles with the 1^st^ statement: "D4 gene is associated with ADHD".*

Auerbach, JG, Benjamin, J, Faroy, M, Geller, V, Ebstein, R (2001) DRD4 related to infant attention and information processing: a developmental link to ADHD? *Psychiatr Genet* **11**: 31-35.

D'Souza, UM *et al* (2004) Functional effects of a tandem duplication polymorphism in the 5'flanking region of the DRD4 gene. *Biol Psychiatry* **56**: 691-697.

Ding, YC *et al* (2002) Evidence of positive selection acting at the human dopamine receptor D4 gene locus. *Proc Natl Acad Sci U S A* **99**: 309-314.

Feng, J *et al* (2001) An in-frame deletion in the alpha(2C) adrenergic receptor is common in African--Americans. *Mol Psychiatry* **6**: 168-172.

Grady, DL *et al* (2003) High prevalence of rare dopamine receptor D4 alleles in children diagnosed with attention-deficit hyperactivity disorder. *Mol Psychiatry* **8**: 536-545.

Hsiung, GY, Kaplan, BJ, Petryshen, TL, Lu, S, Field, LL (2004) A dyslexia susceptibility locus (DYX7) linked to dopamine D4 receptor (DRD4) region on chromosome 11p15.5. *Am J Med Genet B Neuropsychiatr Genet* **125B**: 112-119.

Kramer, UM *et al* (2007) The impact of catechol-O-methyltransferase and dopamine D4 receptor genotypes on neurophysiological markers of performance monitoring. *J Neurosci* **27**: 14190-14198.

Kramer, UM *et al* (2009) ADHD candidate gene (DRD4 exon III) affects inhibitory control in a healthy sample. *BMC Neurosci* **10**: 150.

Lakatos, K *et al* (2003) Association of D4 dopamine receptor gene and serotonin transporter promoter polymorphisms with infants' response to novelty. *Mol Psychiatry* **8**: 90-97.

Laurin, N *et al* (2008) No evidence for genetic association between DARPP-32 (PP1R1B) polymorphisms and attention deficit hyperactivity disorder. *Am J Med Genet B Neuropsychiatr Genet* **147**: 339-342.

Lowe, N *et al* (2004) Joint analysis of the DRD5 marker concludes association with attention-deficit/hyperactivity disorder confined to the predominantly inattentive and combined subtypes. *Am J Hum Genet* **74**: 348-356.

Payton, A *et al* (2001) Susceptibility genes for a trait measure of attention deficit hyperactivity disorder: a pilot study in a non-clinical sample of twins. *Psychiatry Res* **105**: 273-278.

Purper-Ouakil, D *et al* (2005) Meta-analysis of family-based association studies between the dopamine transporter gene and attention deficit hyperactivity disorder. *Psychiatr Genet* **15**: 53-59.

Roman, T *et al* (2003) Is the alpha-2A adrenergic receptor gene (ADRA2A) associated with attention-deficit/hyperactivity disorder? *Am J Med Genet B Neuropsychiatr Genet* **120B**: 116-120.

Rondou, P, Haegeman, G, Vanhoenacker, P, Van Craenenbroeck, K (2008) BTB Protein KLHL12 targets the dopamine D4 receptor for ubiquitination by a Cul3-based E3 ligase. *J Biol Chem* **283**: 11083-11096.

Schmidt, LA, Fox, NA, Perez-Edgar, K, Hu, S, Hamer, DH (2001) Association of DRD4 with attention problems in normal childhood development. *Psychiatr Genet* **11**: 25-29.

Sheese, BE, Voelker, PM, Rothbart, MK, Posner, MI (2007) Parenting quality interacts with genetic variation in dopamine receptor D4 to influence temperament in early childhood. *Dev Psychopathol* **19**: 1039-1046.

Sonuga-Barke, EJ *et al* (2009) Dopamine and serotonin transporter genotypes moderate sensitivity to maternal expressed emotion: the case of conduct and emotional problems in attention deficit/hyperactivity disorder. *J Child Psychol Psychiatry* **50**: 1052-1063.

Szekely, A *et al* (2004) Human personality dimensions of persistence and harm avoidance associated with DRD4 and 5-HTTLPR polymorphisms. *Am J Med Genet B Neuropsychiatr Genet* **126B**: 106-110.

Todd, RD, Neuman, RJ (2007) Gene-environment interactions in the development of combined type ADHD: evidence for a synapse-based model. *Am J Med Genet B Neuropsychiatr Genet* **144B**: 971-975.

Turic, D *et al* (2004) Direct analysis of the genes encoding G proteins G alpha T2, G alpha o, G alpha Z in ADHD. *Am J Med Genet B Neuropsychiatr Genet* **127B**: 68-72.

Van Craenenbroeck, K *et al* (2005) Folding efficiency is rate-limiting in dopamine D4 receptor biogenesis. *J Biol Chem* **280**: 19350-19357.

Wang, E *et al* (2004) The genetic architecture of selection at the human dopamine receptor D4 (DRD4) gene locus. *Am J Hum Genet* **74**: 931-944.

Williams J, Taylor E (2006) The evolution of hyperactivity, impulsivity and cognitive diversity. J R Soc Interface 3:399-413.

Yamagata, T *et al* (2002) The human secretin gene: fine structure in 11p15.5 and sequence variation in patients with autism. *Genomics* **80**: 185-194.

*3.2) Articles not relevant to the present investigation*

Kim B, Koo MS, Jun JY, Park IH, Oh DY, Cheon KA (2009) Association between Dopamine D4 Receptor Gene Polymorphism and Scores on a Continuous Performance Test in Korean Children with Attention Deficit Hyperactivity Disorder. Psychiatry Investig 6:216-221.

**4) Studies in humans that reported data about the DRD4 gene (117)**

*4.1) Articles with the 1^st^ statement: "D4 gene is associated with ADHD".*

Altink, ME *et al* (2008) The dopamine receptor D4 7-repeat allele and prenatal smoking in ADHD-affected children and their unaffected siblings: no gene-environment interaction. *J Child Psychol Psychiatry* **49**: 1053-1060.

Altink, ME *et al* (2009) Effects of maternal and paternal smoking on attentional control in children with and without ADHD. *Eur Child Adolesc Psychiatry*.

Ballon, N *et al* (2007) Polymorphisms TaqI A of the DRD2, BalI of the DRD3, exon III repeat of the DRD4, and 3' UTR VNTR of the DAT: association with childhood ADHD in male African-Caribbean cocaine dependents? *Am J Med Genet B Neuropsychiatr Genet* **144B**: 1034-1041.

Barr, CL *et al* (2000) Further evidence from haplotype analysis for linkage of the dopamine D4 receptor gene and attention-deficit hyperactivity disorder. *Am J Med Genet* **96**: 262-267.

Becker, K, Laucht, M, El-Faddagh, M, Schmidt, MH (2005) The dopamine D4 receptor gene exon III polymorphism is associated with novelty seeking in 15-year-old males from a high-risk community sample. *J Neural Transm* **112**: 847-858.

Bellgrove, MA *et al* (2005) DRD4 gene variants and sustained attention in attention deficit hyperactivity disorder (ADHD): effects of associated alleles at the VNTR and -521 SNP. *Am J Med Genet B Neuropsychiatr Genet* **136B**: 81-86.

Bhaduri, N *et al* (2006) Association of dopamine D4 receptor (DRD4) polymorphisms with attention deficit hyperactivity disorder in Indian population. *Am J Med Genet B Neuropsychiatr Genet* **141B**: 61-66.

Biederman, J *et al* (2009) Effect of candidate gene polymorphisms on the course of attention deficit hyperactivity disorder. *Psychiatry Res*.

Brookes, K *et al* (2006) The analysis of 51 genes in DSM-IV combined type attention deficit hyperactivity disorder: association signals in DRD4, DAT1 and 16 other genes. *Mol Psychiatry* **11**: 934-953.

Brookes, KJ *et al* (2008) Differential dopamine receptor D4 allele association with ADHD dependent of proband season of birth. *Am J Med Genet B Neuropsychiatr Genet* **147B**: 94-99.

Carrasco, X *et al* (2006) Genotypic interaction between DRD4 and DAT1 loci is a high risk factor for attention-deficit/hyperactivity disorder in Chilean families. *Am J Med Genet B Neuropsychiatr Genet* **141B**: 51-54.

Cheon, KA, Kim, BN, Cho, SC (2007) Association of 4-repeat allele of the dopamine D4 receptor gene exon III polymorphism and response to methylphenidate treatment in Korean ADHD children. *Neuropsychopharmacology* **32**: 1377-1383.

Demiralp, T *et al* (2007) DRD4 and DAT1 polymorphisms modulate human gamma band responses. *Cereb Cortex* **17**: 1007-1019.

DeYoung, CG *et al* (2006) The dopamine D4 receptor gene and moderation of the association between externalizing behavior and IQ. *Arch Gen Psychiatry* **63**: 1410-1416.

El-Faddagh, M, Laucht, M, Maras, A, Vohringer, L, Schmidt, MH (2004) Association of dopamine D4 receptor (DRD4) gene with attention-deficit/hyperactivity disorder (ADHD) in a high-risk community sample: a longitudinal study from birth to 11 years of age. *J Neural Transm* **111**: 883-889.

Faraone, SV *et al* (1999) Dopamine D4 gene 7-repeat allele and attention deficit hyperactivity disorder. *Am J Psychiatry* **156**: 768-770.

Gizer, IR *et al* (2008) Relations between multi-informant assessments of ADHD symptoms, DAT1, and DRD4. *J Abnorm Psychol* **117**: 869-880.

Grady, DL *et al* (2005) Sequence variants of the DRD4 gene in autism: further evidence that rare DRD4 7R haplotypes are ADHD specific. *Am J Med Genet B Neuropsychiatr Genet* **136B**: 33-35.

Hamarman, S, Fossella, J, Ulger, C, Brimacombe, M, Dermody, J (2004) Dopamine receptor 4 (DRD4) 7-repeat allele predicts methylphenidate dose response in children with attention deficit hyperactivity disorder: a pharmacogenetic study. *J Child Adolesc Psychopharmacol* **14**: 564-574.

Hawi, Z *et al* (2005) Preferential transmission of paternal alleles at risk genes in attention-deficit/hyperactivity disorder. *Am J Hum Genet* **77**: 958-965.

Holmes, J *et al* (2002) Association of DRD4 in children with ADHD and comorbid conduct problems. *Am J Med Genet* **114**: 150-153.

Johnson, KA *et al* (2008) Absence of the 7-repeat variant of the DRD4 VNTR is associated with drifting sustained attention in children with ADHD but not in controls. *Am J Med Genet B Neuropsychiatr Genet* **147B**: 927-937.

Kieling, C, Roman, T, Doyle, AE, Hutz, MH, Rohde, LA (2006) Association between DRD4 gene and performance of children with ADHD in a test of sustained attention. *Biol Psychiatry* **60**: 1163-1165.

Kirley, A *et al* (2004) Phenotype studies of the DRD4 gene polymorphisms in ADHD: association with oppositional defiant disorder and positive family history. *Am J Med Genet B Neuropsychiatr Genet* **131B**: 38-42.

Kooij, JS *et al* (2008) Response to methylphenidate in adults with ADHD is associated with a polymorphism in SLC6A3 (DAT1). *Am J Med Genet B Neuropsychiatr Genet* **147B**: 201-208.

LaHoste, GJ *et al* (1996) Dopamine D4 receptor gene polymorphism is associated with attention deficit hyperactivity disorder. *Mol Psychiatry* **1**: 121-124.

Langley, K *et al* (2009) Molecular genetic contribution to the developmental course of attention-deficit hyperactivity disorder. *Eur Child Adolesc Psychiatry* **18**: 26-32.

Langley, K *et al* (2004) Association of the dopamine D4 receptor gene 7-repeat allele with neuropsychological test performance of children with ADHD. *Am J Psychiatry* **161**: 133-138.

Langley, K *et al* (2008) Testing for gene x environment interaction effects in attention deficit hyperactivity disorder and associated antisocial behavior. *Am J Med Genet B Neuropsychiatr Genet* **147B**: 49-53.

Lasky-Su, J *et al* (2007) Partial replication of a DRD4 association in ADHD individuals using a statistically derived quantitative trait for ADHD in a family-based association test. *Biol Psychiatry* **62**: 985-990.

Lasky-Su, J *et al* (2008) Family-based association analysis of a statistically derived quantitative traits for ADHD reveal an association in DRD4 with inattentive symptoms in ADHD individuals. *Am J Med Genet B Neuropsychiatr Genet* **147B**: 100-106.

Laucht, M, Hohm, E, Esser, G, Schmidt, MH, Becker, K (2007) Association between ADHD and smoking in adolescence: shared genetic, environmental and psychopathological factors. *J Neural Transm* **114**: 1097-1104.

Levitan, RD *et al* (2004) Childhood inattention and dysphoria and adult obesity associated with the dopamine D4 receptor gene in overeating women with seasonal affective disorder. *Neuropsychopharmacology* **29**: 179-186.

Loo, SK *et al* (2008) Cognitive functioning in affected sibling pairs with ADHD: familial clustering and dopamine genes. *J Child Psychol Psychiatry* **49**: 950-957.

Lynn, DE *et al* (2005) Temperament and character profiles and the dopamine D4 receptor gene in ADHD. *Am J Psychiatry* **162**: 906-913.

McCracken, JT *et al* (2000) Evidence for linkage of a tandem duplication polymorphism upstream of the dopamine D4 receptor gene (DRD4) with attention deficit hyperactivity disorder (ADHD). *Mol Psychiatry* **5**: 531-536.

McGough, J *et al* (2006) Pharmacogenetics of methylphenidate response in preschoolers with ADHD. *J Am Acad Child Adolesc Psychiatry* **45**: 1314-1322.

Mill, J *et al* (2006) Prediction of heterogeneity in intelligence and adult prognosis by genetic polymorphisms in the dopamine system among children with attention-deficit/hyperactivity disorder: evidence from 2 birth cohorts. *Arch Gen Psychiatry* **63**: 462-469.

Monuteaux, MC *et al* (2008) A preliminary study of dopamine D4 receptor genotype and structural brain alterations in adults with ADHD. *Am J Med Genet B Neuropsychiatr Genet* **147B**: 1436-1441.

Muglia, P, Jain, U, Macciardi, F, Kennedy, JL (2000) Adult attention deficit hyperactivity disorder and the dopamine D4 receptor gene. *Am J Med Genet* **96**: 273-277.

Neuman, RJ *et al* (2007) Prenatal smoking exposure and dopaminergic genotypes interact to cause a severe ADHD subtype. *Biol Psychiatry* **61**: 1320-1328.

Rowe, DC *et al* (2001) Two dopamine genes related to reports of childhood retrospective inattention and conduct disorder symptoms. *Mol Psychiatry* **6**: 429-433.

Rowe, DC *et al* (1998) Dopamine DRD4 receptor polymorphism and attention deficit hyperactivity disorder. *Mol Psychiatry* **3**: 419-426.

Seeger, G, Schloss, P, Schmidt, MH, Ruter-Jungfleisch, A, Henn, FA (2004) Gene-environment interaction in hyperkinetic conduct disorder (HD + CD) as indicated by season of birth variations in dopamine receptor (DRD4) gene polymorphism. *Neurosci Lett* **366**: 282-286.

Shaw, P *et al* (2007) Polymorphisms of the dopamine D4 receptor, clinical outcome, and cortical structure in attention-deficit/hyperactivity disorder. *Arch Gen Psychiatry* **64**: 921-931.

Sonuga-Barke, EJ *et al* (2008) Intelligence in DSM-IV combined type attention-deficit/hyperactivity disorder is not predicted by either dopamine receptor/transporter genes or other previously identified risk alleles for attention-deficit/hyperactivity disorder. *Am J Med Genet B Neuropsychiatr Genet* **147**: 316-319.

Sunohara, GA *et al* (2000) Linkage of the dopamine D4 receptor gene and attention-deficit/hyperactivity disorder. *J Am Acad Child Adolesc Psychiatry* **39**: 1537-1542.

Swanson, J *et al* (2000) Attention deficit/hyperactivity disorder children with a 7-repeat allele of the dopamine receptor D4 gene have extreme behavior but normal performance on critical neuropsychological tests of attention. *Proc Natl Acad Sci U S A* **97**: 4754-4759.

Swanson, JM *et al* (2007) Effects of source of DNA on genotyping success rates and allele percentages in the Preschoolers with Attention-Deficit/Hyperactivity Disorder Treatment Study (PATS). *J Child Adolesc Psychopharmacol* **17**: 635-646.

Szobot, C *et al* (2005) Brain perfusion and dopaminergic genes in boys with attention-deficit/hyperactivity disorder. *Am J Med Genet B Neuropsychiatr Genet* **132B**: 53-58.

Tahir, E *et al* (2000) Association and linkage of DRD4 and DRD5 with attention deficit hyperactivity disorder (ADHD) in a sample of Turkish children. *Mol Psychiatry* **5**: 396-404.

Tharoor, H, Lobos, EA, Todd, RD, Reiersen, AM (2008) Association of dopamine, serotonin, and nicotinic gene polymorphisms with methylphenidate response in ADHD. *Am J Med Genet B Neuropsychiatr Genet* **147B**: 527-530.

van der Meulen, EM *et al* (2005) High sibling correlation on methylphenidate response but no association with DAT1-10R homozygosity in Dutch sibpairs with ADHD. *J Child Psychol Psychiatry* **46**: 1074-1080.

Waldman, ID (2005) Statistical approaches to complex phenotypes: evaluating neuropsychological endophenotypes for attention-deficit/hyperactivity disorder. *Biol Psychiatry* **57**: 1347-1356.

Yang, JW *et al* (2008) A case-control association study of the polymorphism at the promoter region of the DRD4 gene in Korean boys with attention deficit-hyperactivity disorder: evidence of association with the -521 C/T SNP. *Prog Neuropsychopharmacol Biol Psychiatry* **32**: 243-248.

*4.2) Articles with the 2^nd^ statement: "D4 gene is associated but it confers small risk"*

Arcos-Burgos, M *et al* (2004) Pedigree disequilibrium test (PDT) replicates association and linkage between DRD4 and ADHD in multigenerational and extended pedigrees from a genetic isolate. *Mol Psychiatry* **9**: 252-259.

Curran, S *et al* (2001) QTL association analysis of the DRD4 exon 3 VNTR polymorphism in a population sample of children screened with a parent rating scale for ADHD symptoms. *Am J Med Genet* **105**: 387-393.

Durston, S *et al* (2005) Differential effects of DRD4 and DAT1 genotype on fronto-striatal gray matter volumes in a sample of subjects with attention deficit hyperactivity disorder, their unaffected siblings, and controls. *Mol Psychiatry* **10**: 678-685.

Faraone, SV, Doyle, AE, Mick, E, Biederman, J (2001) Meta-analysis of the association between the 7-repeat allele of the dopamine D(4) receptor gene and attention deficit hyperactivity disorder. *Am J Psychiatry* **158**: 1052-1057.

Gornick, MC *et al* (2007) Association of the dopamine receptor D4 (DRD4) gene 7-repeat allele with children with attention-deficit/hyperactivity disorder (ADHD): an update. *Am J Med Genet B Neuropsychiatr Genet* **144B**: 379-382.

Holmes, J *et al* (2000) A family-based and case-control association study of the dopamine D4 receptor gene and dopamine transporter gene in attention deficit hyperactivity disorder. *Mol Psychiatry* **5**: 523-530.

Kereszturi, E *et al* (2007) Association between the 120-bp duplication of the dopamine D4 receptor gene and attention deficit hyperactivity disorder: genetic and molecular analyses. *Am J Med Genet B Neuropsychiatr Genet* **144B**: 231-236.

Kustanovich, V *et al* (2004) Transmission disequilibrium testing of dopamine-related candidate gene polymorphisms in ADHD: confirmation of association of ADHD with DRD4 and DRD5. *Mol Psychiatry* **9**: 711-717.

Leung, PW *et al* (2005) Dopamine receptor D4 (DRD4) gene in Han Chinese children with attention-deficit/hyperactivity disorder (ADHD): increased prevalence of the 2-repeat allele. *Am J Med Genet B Neuropsychiatr Genet* **133B**: 54-56.

Li, D, Sham, PC, Owen, MJ, He, L (2006) Meta-analysis shows significant association between dopamine system genes and attention deficit hyperactivity disorder (ADHD). *Hum Mol Genet* **15**: 2276-2284.

Lowe, N *et al* (2004) Multiple marker analysis at the promoter region of the DRD4 gene and ADHD: evidence of linkage and association with the SNP -616. *Am J Med Genet B Neuropsychiatr Genet* **131B**: 33-37.

Maher, BS, Marazita, ML, Ferrell, RE, Vanyukov, MM (2002) Dopamine system genes and attention deficit hyperactivity disorder: a meta-analysis. *Psychiatr Genet* **12**: 207-215.

McClernon, FJ, Fuemmeler, BF, Kollins, SH, Kail, ME, Ashley-Koch, AE (2008) Interactions between genotype and retrospective ADHD symptoms predict lifetime smoking risk in a sample of young adults. *Nicotine Tob Res* **10**: 117-127.

Mill, J *et al* (2001) Attention deficit hyperactivity disorder (ADHD) and the dopamine D4 receptor gene: evidence of association but no linkage in a UK sample. *Mol Psychiatry* **6**: 440-444.

Oades, RD *et al* (2008) The influence of serotonin- and other genes on impulsive behavioral aggression and cognitive impulsivity in children with attention-deficit/hyperactivity disorder (ADHD): Findings from a family-based association test (FBAT) analysis. *Behav Brain Funct* **4**: 48.

Qian, Q *et al* (2007) Evaluation of potential gene-gene interactions for attention deficit hyperactivity disorder in the Han Chinese population. *Am J Med Genet B Neuropsychiatr Genet* **144B**: 200-206.

Roman, T *et al* (2001) Attention-deficit hyperactivity disorder: a study of association with both the dopamine transporter gene and the dopamine D4 receptor gene. *Am J Med Genet* **105**: 471-478.

Smalley, SL *et al* (1998) Evidence that the dopamine D4 receptor is a susceptibility gene in attention deficit hyperactivity disorder. *Mol Psychiatry* **3**: 427-430.

Swanson, JM *et al* (1998) Association of the dopamine receptor D4 (DRD4) gene with a refined phenotype of attention deficit hyperactivity disorder (ADHD): a family-based approach. *Mol Psychiatry* **3**: 38-41.

*4.3) Articles with the 3^rd^ statement: "D4 is not associated with ADHD.*

Bakker, SC *et al* (2005) DAT1, DRD4, and DRD5 polymorphisms are not associated with ADHD in Dutch families. *Am J Med Genet B Neuropsychiatr Genet* **132B**: 50-52.

Barkley, RA, Smith, KM, Fischer, M, Navia, B (2006) An examination of the behavioral and neuropsychological correlates of three ADHD candidate gene polymorphisms (DRD4 7+, DBH TaqI A2, and DAT1 40 bp VNTR) in hyperactive and normal children followed to adulthood. *Am J Med Genet B Neuropsychiatr Genet* **141B**: 487-498.

Barr, CL *et al* (2001) 5'-untranslated region of the dopamine D4 receptor gene and attention-deficit hyperactivity disorder. *Am J Med Genet* **105**: 84-90.

Bobb, AJ *et al* (2005) Support for association between ADHD and two candidate genes: NET1 and DRD1. *Am J Med Genet B Neuropsychiatr Genet* **134B**: 67-72.

Brookes, KJ *et al* (2005) No evidence for the association of DRD4 with ADHD in a Taiwanese population within-family study. *BMC Med Genet* **6**: 31.

Castellanos, FX *et al* (1998) Lack of an association between a dopamine-4 receptor polymorphism and attention-deficit/hyperactivity disorder: genetic and brain morphometric analyses. *Mol Psychiatry* **3**: 431-434.

Cheuk, DK, Li, SY, Wong, V (2006) Exon 3 polymorphisms of dopamine D4 receptor (DRD4) gene and attention deficit hyperactivity disorder in Chinese children. *Am J Med Genet B Neuropsychiatr Genet* **141B**: 907-911.

Comings, DE *et al* (1999) Studies of the 48 bp repeat polymorphism of the DRD4 gene in impulsive, compulsive, addictive behaviors: Tourette syndrome, ADHD, pathological gambling, and substance abuse. *Am J Med Genet* **88**: 358-368.

Eisenberg, J *et al* (2000) A haplotype relative risk study of the dopamine D4 receptor (DRD4) exon III repeat polymorphism and attention deficit hyperactivity disorder (ADHD). *Am J Med Genet* **96**: 258-261.

Fisher, SE *et al* (2002) A genomewide scan for loci involved in attention-deficit/hyperactivity disorder. *Am J Hum Genet* **70**: 1183-1196.

Frank, Y, Pergolizzi, RG, Perilla, MJ (2004) Dopamine D4 receptor gene and attention deficit hyperactivity disorder. *Pediatr Neurol* **31**: 345-348.

Gabriela, ML *et al* (2009) Genetic interaction analysis for DRD4 and DAT1 genes in a group of Mexican ADHD patients. *Neurosci Lett* **451**: 257-260.

Hawi, Z *et al* (2000) No association of the dopamine DRD4 receptor (DRD4) gene polymorphism with attention deficit hyperactivity disorder (ADHD) in the Irish population. *Am J Med Genet* **96**: 268-272.

Johansson, S *et al* (2008) Genetic analyses of dopamine related genes in adult ADHD patients suggest an association with the DRD5-microsatellite repeat, but not with DRD4 or SLC6A3 VNTRs. *Am J Med Genet B Neuropsychiatr Genet* **147B**: 1470-1475.

Kereszturi, E *et al* (2008) Catechol-O-methyltransferase Val158Met polymorphism is associated with methylphenidate response in ADHD children. *Am J Med Genet B Neuropsychiatr Genet* **147B**: 1431-1435.

Kim, YS *et al* (2005) Family-based association study of DAT1 and DRD4 polymorphism in Korean children with ADHD. *Neurosci Lett* **390**: 176-181.

Kotler, M *et al* (2000) Failure to replicate an excess of the long dopamine D4 exon III repeat polymorphism in ADHD in a family-based study. *Am J Med Genet* **96**: 278-281.

Manor, I *et al* (2002) The short DRD4 repeats confer risk to attention deficit hyperactivity disorder in a family-based design and impair performance on a continuous performance test (TOVA). *Mol Psychiatry* **7**: 790-794.

Mill, J *et al* (2003) Polymorphisms in the dopamine D4 receptor gene and attention-deficit hyperactivity disorder. *Neuroreport* **14**: 1463-1466.

Mill, J *et al* (2005) Quantitative trait locus analysis of candidate gene alleles associated with attention deficit hyperactivity disorder (ADHD) in five genes: DRD4, DAT1, DRD5, SNAP-25, and 5HT1B. *Am J Med Genet B Neuropsychiatr Genet* **133B**: 68-73.

Mill, JS *et al* (2002) The dopamine D4 receptor and the hyperactivity phenotype: a developmental-epidemiological study. *Mol Psychiatry* **7**: 383-391.

Niederhofer, H *et al* (2008) A preliminary report of the dopamine receptor D(4) and the dopamine transporter 1 gene polymorphism and its association with attention deficit hyperactivity disorder. *Neuropsychiatr Dis Treat* **4**: 701-705.

Payton, A *et al* (2001) Examining for association between candidate gene polymorphisms in the dopamine pathway and attention-deficit hyperactivity disorder: a family-based study. *Am J Med Genet* **105**: 464-470.

Qian, Q, Wang, Y, Zhou, R, Yang, L, Faraone, SV (2004) Family-based and case-control association studies of DRD4 and DAT1 polymorphisms in Chinese attention deficit hyperactivity disorder patients suggest long repeats contribute to genetic risk for the disorder. *Am J Med Genet B Neuropsychiatr Genet* **128B**: 84-89.

Smith, KM *et al* (2003) Association of the dopamine beta hydroxylase gene with attention deficit hyperactivity disorder: genetic analysis of the Milwaukee longitudinal study. *Am J Med Genet B Neuropsychiatr Genet* **119B**: 77-85.

Todd, RD *et al* (2005) Collaborative analysis of DRD4 and DAT genotypes in population-defined ADHD subtypes. *J Child Psychol Psychiatry* **46**: 1067-1073.

Todd, RD *et al* (2001) Lack of association of dopamine D4 receptor gene polymorphisms with ADHD subtypes in a population sample of twins. *Am J Med Genet* **105**: 432-438.

*4.4) Articles not relevant to the present investigation.*

Bakermans-Kranenburg, MJ, Van, IMH, Pijlman, FT, Mesman, J, Juffer, F (2008) Experimental evidence for differential susceptibility: dopamine D4 receptor polymorphism (DRD4 VNTR) moderates intervention effects on toddlers' externalizing behavior in a randomized controlled trial. *Dev Psychol* **44**: 293-300.

Boonstra, AM *et al* (2008) An exploratory study of the relationship between four candidate genes and neurocognitive performance in adult ADHD. *Am J Med Genet B Neuropsychiatr Genet* **147**: 397-402.

Diaz-Anzaldua, A *et al* (2004) Tourette syndrome and dopaminergic genes: a family-based association study in the French Canadian founder population. *Mol Psychiatry* **9**: 272-277.

Ettinger, U, Joober, R, R, DEG, O'Driscoll G, A (2006) Schizotypy, attention deficit hyperactivity disorder, and dopamine genes. *Psychiatry Clin Neurosci* **60**: 764-767.

Kebir, O, Grizenko, N, Sengupta, S, Joober, R (2009) Verbal but not performance IQ is highly correlated to externalizing behavior in boys with ADHD carrying both DRD4 and DAT1 risk genotypes. *Prog Neuropsychopharmacol Biol Psychiatry* **33**: 939-944.

Lasky-Su, J *et al* (2007) Evidence for an association of the dopamine D5 receptor gene on age at onset of attention deficit hyperactivity disorder. *Ann Hum Genet* **71**: 648-659.

Leddy, JJ *et al* (2009) Dopamine-related genotypes and the dose-response effect of methylphenidate on eating in attention-deficit/hyperactivity disorder youths. *J Child Adolesc Psychopharmacol* **19**: 127-136.

Marino, C *et al* (2003) No evidence for association and linkage disequilibrium between dyslexia and markers of four dopamine-related genes. *Eur Child Adolesc Psychiatry* **12**: 198-202.

McGough, JJ *et al* (2009) A Candidate Gene Analysis of Methylphenidate Response in Attention-Deficit/Hyperactivity Disorder. *J Am Acad Child Adolesc Psychiatry*.

Nigg, J, Nikolas, M, Friderici, K, Park, L, Zucker, RA (2007) Genotype and neuropsychological response inhibition as resilience promoters for attention-deficit/hyperactivity disorder, oppositional defiant disorder, and conduct disorder under conditions of psychosocial adversity. *Dev Psychopathol* **19**: 767-786.

Seeger, G, Schloss, P, Schmidt, MH (2001) Marker gene polymorphisms in hyperkinetic disorder--predictors of clinical response to treatment with methylphenidate? *Neurosci Lett* **313**: 45-48.

Stevens, SE *et al* (2009) Dopamine transporter gene polymorphism moderates the effects of severe deprivation on ADHD symptoms: developmental continuities in gene-environment interplay. *Am J Med Genet B Neuropsychiatr Genet* **150B**: 753-761.

Volk, HE, Todd, RD (2007) Does the Child Behavior Checklist juvenile bipolar disorder phenotype identify bipolar disorder? *Biol Psychiatry* **62**: 115-120.

Winsberg, BG, Comings, DE (1999) Association of the dopamine transporter gene (DAT1) with poor methylphenidate response. *J Am Acad Child Adolesc Psychiatry* **38**: 1474-1477.

Yoon, DY *et al* (2007) Dopaminergic polymorphisms in Tourette syndrome: association with the DAT gene (SLC6A3). *Am J Med Genet B Neuropsychiatr Genet* **144B**: 605-610.

Zeni, CP *et al* (2007) No significant association between response to methylphenidate and genes of the dopaminergic and serotonergic systems in a sample of Brazilian children with attention-deficit/hyperactivity disorder. *Am J Med Genet B Neuropsychiatr Genet* **144B**: 391-394.

**B) Media articles reporting on the association between alleles of the gene coding for the D4 dopamine receptor and ADHD.**

*1) Articles stating that the DRD4 gene is associated with ADHD without mitigating comment*

Maybe it's not a gene behind a person's thrill-seeking ways. November 1st, 1996. *The New York Times.*

Doubt cast on gene linked to behaviour novelty-seeking trait not determined by genetics in U.S., Finnish studies. November 6, 1996. *The Globe and Mail.*

Variants, the spice of life. May 19, 1998. *The Independent - London.*

Hyperactivity - a rational strategy. September 1st, 1999. *The Practitioner.*

Monitoring Kids' Attention / We may be overdiagnosing the deficit disorder ADD. October 10, 1999. *Newsday.*

Mental illness: studies highlight molecular basis of disorders in children. September 22, 2000. *Genomics & Genetics Weekly.*

Mental illness: studies highlight molecular basis of disorders in children. September 23, 2000. *Pain & Central Nervous System Week.*

Knowledge of attention deficit continues to grow. September 5, 2002. *Calgary Herald.*

ADHD gene find. January 8, 2002. *Associated Press Newswires.*

Attention-Deficit Hyperactivity Disorder related to advantageous gene. January 8, 2002. *Ascribe News.*

UCI researchers find genetic link for ADHD. January 2002, 2002. *The Orange County Register.*

A game of risk. August 17, 2002. *The Globe and Mail.*

Role of dopamine D4 receptor gene mutations in ADHD clarified. October 27, 2003. *Mental Health Weekly Digest.*

Role of dopamine D4 receptor gene mutations in ADHD clarified. October 27, 2003. *Pain & Central Nervous System Week.*

Role of dopamine D4 receptor gene mutations in ADHD clarified. October 27, 2003. *Health & Medicine Week.*

Institute of Psychiatry, London; Role of dopamine D4 receptor gene mutations in ADHD clarified. October 29, 2003. *Biotech Week.*

Neuroscience; Role of dopamine D4 receptor gene mutations in ADHD clarified. October 31, 2003. *Genomics & Genetics Weekly.*

Is it ADHD that's eating the boss? September 15, 2004. *The Times.*

Birth season affects gene linkage to hyperkinetic and conduct disorders. October 25, 2004. *Health & Medicine Week.*

Behavioral Genetics; Birth season affects gene linkage to hyperkinetic and conduct disorders. October 25, 2004. *Mental Health Weekly Digest.*

Birth season affects gene linkage to hyperkinetic and conduct disorders. October 26, 2004. *Life Science Weekly.*

Birth season affects gene linkage to hyperkinetic and conduct disorders. October 26, 2004. *Science Letter.*

Central Institute of Mental Health Mannheim; Birth season affects gene linkage to hyperkinetic and conduct disorders. October 27, 2004. *Biotech Week.*

Behavioral Genetics; Birth season affects gene linkage to hyperkinetic and conduct disorders. October 29, 2004. *Drug Week.*

Birth season affects gene linkage to hyperkinetic and conduct disorders. October 29, 2004. *Genomics & Genetics Weekly.*

Birth season affects gene linkage to hyperkinetic and conduct disorders. October 30, 2004. *Obesity, Fitness & Wellness Week.*

DRD4 gene polymorphisms associate with attention deficit hyperactivity disorder. January 3, 2005. *Mental Health Weekly Digest.*

Genetic Disorders; DRD4 gene polymorphisms associate with attention deficit hyperactivity disorder. Janaury 3, 2005. *Health & Medicine Week.*

DRD4 gene polymorphisms associate with attention deficit hyperactivity disorder. January 4, 2005. *Life Science Weekly.*

DRD4 gene polymorphisms associate with attention deficit hyperactivity disorder. January 4, 2005. *Science Letter.*

University of Dublin Trinity College; DRD4 gene polymorphisms associate with attention deficit hyperactivity disorder. January 5, 2005. *Biotech Week.*

DRD4 gene polymorphisms associate with attention deficit hyperactivity disorder. January 7, 2005. *Drug Week.*

DRD4 gene polymorphisms associate with attention deficit hyperactivity disorder. January 8, 2005. *Obesity, Fitness & Wellness Week.*

Genetic Disorders; DRD4 gene polymorphisms associate with attention deficit hyperactivity disorder. January 7, 2005. *Genomics & Genetics Weekly.*

And us; Some say it has stopped, others believe it's going faster than ever. So which is it, asks Kate Douglas. March 11, 2006. *New Scientist.*

Lead exposure causes Attention-Deficit Hyperactivity Disorder among children. May 4, 2006. *Pakistan Press International Information Services.*

Study links ADHD cognitive and behavioral problems to gene and environment. May 22, 2006. *Biotech Week.*

Attention Deficit Hyperactivity Disorder; Study links ADHD cognitive and behavioral problems to gene and environment. May 22, 2006. *Mental Health Weekly Digest.*

Study links ADHD cognitive and behavioral problems to gene and environment. May 23, 2006. *Obesity, Fitness & Wellness Week.*

Attention Deficit Hyperactivity Disorder; Study links ADHD cognitive and behavioral problems to gene and environment. May 24, 2006. *Life Science Weekly.*

Attention Deficit Hyperactivity Disorder; Study links ADHD cognitive and behavioral problems to gene and environment. May 25, 2006. *Health & Medicine Week.*

Study links ADHD cognitive and behavioral problems to gene and environment. May 26, 2006. *Genomics & Genetics Weekly.*

Study links ADHD cognitive and behavioral problems to gene and environment. May 26, 2006. *Science Letter.*

Researchers at Institute of Psychiatry publish new data on attention deficit hyperactivity disorder. October 30, 2006. *Mental Health Weekly Digest.*

University of Rio Grande, Department of Psychiatry publishes research in attention deficit hyperactivity disorder. December 11, 2006. *Mental Health Weekly Digest.*

Attention Deficit Hyperactivity Disorder; Gene variant is associated with brain anatomy, clinical course of ADHD. August 21, 2007. *Life Science Weekly.*

Why some children respond better to parenting. March 21, 2007. *Hindustan Times.*

Reports summarize mental health research from Peking University, Institute of Mental Health. April 9, 2007. *Mental Health Weekly Digest.*

Reports summarize mental health research from Peking University, Institute of Mental Health. April 9, 2007. *Biotech Business Week.*

Reports summarize mental health research from Peking University, Institute of Mental Health. April 9, 2007. *Pharma Business Week.*

Reports summarize mental health research from Peking University, Institute of Mental Health. April 13, 2007. *Drug Week.*

Reports summarize mental health research from Peking University, Institute of Mental Health. April 14, 2007. *Obesity, Fitness & Wellness Week.*

Research from Peking University, People's Republic of China, provides new insights into human health. May 8, 2007. *Science Letter.*

Reports outline life sciences study findings from Istanbul University, Istanbul Faculty of Medicine. May 14, 2007. *Biotech Business Week.*

Vulnerable brains. May 17, 2007. *Hospital Doctor.*

Reports outline life sciences study findings from Istanbul University, Istanbul Faculty of Medicine. May 18, 2007. *Drug Week.*

Prenatal exposure to smoking increases ADHD risk in children. May 24, 2007. *Asian News International.*

Gene Variant Is Associated With Brain Anatomy, Clinical Course of ADHD. August 20, 2007. *Biotech Business Week.*

Attention deficit hyperactivity disorder; Gene variant is associated with brain anatomy, clinical course of ADHD. August 20, 2007. *Health & Medicine Week.*

Gene variant is associated with brain anatomy, clinical course of ADHD. August 20, 2007. *Mental Health Weekly Digest.*

Attention Deficit Hyperactivity Disorder; Gene variant is associated with brain anatomy, clinical course of ADHD. August 20, 2007. *Pharma Business Week.*

Attention Deficit Hyperactivity Disorder; Gene variant is associated with brain anatomy, clinical course of ADHD. August 21, 2007. *Science Letter.*

Attention Deficit Hyperactivity Disorder; Gene variant is associated with brain anatomy, clinical course of ADHD. August 22, 2007. *Biotech Week.*

Attention Deficit Hyperactivity Disorder; Gene variant is associated with brain anatomy, clinical course of ADHD. August 24, 2007. *Drug Week.*

Attention Deficit Hyperactivity Disorder; Gene variant is associated with brain anatomy, clinical course of ADHD. August 25, 2007. *Obesity, Fitness & Wellness Week.*

National Institute of Mental Health reports research in attention deficit hyperactivity disorder therapy. September 3, 2007. *Biotech Business Week.*

National Institute of Mental Health reports research in attention deficit hyperactivity disorder therapy. September 3, 2007. *Mental Health Weekly Digest.*

National Institute of Mental Health reports research in attention deficit hyperactivity disorder therapy. September 3, 2007. *Health & Medicine Week.*

National Institute of Mental Health reports research in attention deficit hyperactivity disorder therapy. September 3, 2007. *Pharma Business Week.*

National Institute of Mental Health reports research in attention deficit hyperactivity disorder therapy. September 4, 2007. *Life Science Weekly.*

National Institute of Mental Health reports research in attention deficit hyperactivity disorder therapy. September 4, 2007. *Science Letter.*

National Institute of Mental Health reports research in attention deficit hyperactivity disorder therapy. September 5, 2007. *Biotech Week.*

National Institute of Mental Health reports research in attention deficit hyperactivity disorder therapy. September 7, 2007. *Drug Week.*

National Institute of Mental Health reports research in attention deficit hyperactivity disorder therapy. September 8, 2007. *Obesity, Fitness & Wellness Week.*

PET scans show depressed dopamine activity in ADHD. October 1st, 2007. *Clinical Psychiatry News.*

Data on attention deficit hyperactivity disorder detailed by researchers at Upstate Medical University, Department of Psychiatry. November 26, 2007. *Mental Health Weekly Digest.*

Data on attention deficit hyperactivity disorder detailed by researchers at Upstate Medical University, November 26, 2007. *Health & Medicine Week.*

Data on attention deficit hyperactivity disorder detailed by researchers at Upstate Medical University, Department of Psychiatry. November 27, 2007. *Life Science Weekly.*

Data on attention deficit hyperactivity disorder detailed by researchers at Upstate Medical University, Department of Psychiatry. November 27, 2007. *Science Letter.*

Data on attention deficit hyperactivity disorder detailed by researchers at Upstate Medical University, Department of Psychiatry. November 28, 2007. *Biotech Week.*

Data on attention deficit hyperactivity disorder detailed by researchers at Upstate Medical University, Department of Psychiatry. December 1st, 2007. *Obesity, Fitness & Wellness Week.*

Reports outline life sciences study findings from Istanbul University, Istanbul Faculty of Medicine. May 14, 2007. *Pharma Business Week.*

Change our schools, not our children. March 15, 2007. *The Independent.*

Findings in mental health reported from Cardiff University, Department of Psychological Medicine. January 7, 2008. *Health & Medicine Week.*

Findings in mental health reported from Cardiff University, Department of Psychological Medicine. January 7, 2008. *Biotech Business Week.*

Findings in mental health reported from Cardiff University, Department of Psychological Medicine. January 7, 2008. *Pharma Business Week.*

Findings in mental health reported from Cardiff University, Department of Psychological Medicine. January 7, 2008. *Mental Health Weekly Digest.*

Findings in mental health reported from Cardiff University, Department of Psychological Medicine. January 9, 2008. *Biotech Week.*

Findings in mental health reported from Cardiff University, Department of Psychological Medicine. January 11, 2008. *Drug Week.*

Findings in mental health reported from Cardiff University, Department of Psychological Medicine. January 12, 2008. *Obesity, Fitness & Wellness Week.*

Researchers at Semmelweis University target attention deficit hyperactivity disorder. January 28, 2008. *Biotech Business Week.*

Researchers at Semmelweis University target attention deficit hyperactivity disorder. January 28, 2008. *Mental Health Weekly Digest.*

Researchers at Semmelweis University target attention deficit hyperactivity disorder. January 28, 2008. *Pharma Business Week.*

Researchers at Semmelweis University target attention deficit hyperactivity disorder. January 28, 2008. *Health & Medicine Week.*

Researchers at Semmelweis University target attention deficit hyperactivity disorder. January 30, 2008. *Biotech Week.*

Researchers at Semmelweis University target attention deficit hyperactivity disorder. February 1st, 2008. *Drug Week.*

Researchers at Semmelweis University target attention deficit hyperactivity disorder. February 2, 2008. *Obesity, Fitness & Wellness Week.*

Research results from Leiden University update knowledge of behavior in children. February 12, 2008. *Life Science Weekly.*

Research results from Leiden University update knowledge of behavior in children. February 15, 2008. *Drug Week.*

Research results from Leiden University update knowledge of behavior in children. February 16, 2008. *Obesity, Fitness & Wellness Week.*

New physiology study findings have been published by scientists at Otto-von-Guericke University. March 29, 2008. *Obesity, Fitness & Wellness Week.*

New findings from Sungkyunkwan University, Department of Psychiatry in the area of attention deficit hyperactivity disorder published. March 29, 2008. *Obesity, Fitness & Wellness Week.*

New findings from Duke University in the area of ADHD risk factors published. April 19, 2008. *Obesity, Fitness & Wellness Week.*

Did the gene for adhd help our nomadic ancestors? June 4, 2008. *Targeted News Service.*

Recent findings from Ghent University highlight research in life sciences. June 7, 2008. *Obesity, Fitness & Wellness Week.*

The misfits - Evolution and genetics. June 14, 2008. *The Economist.*

ADHD an advantage for nomadic tribesmen? June 28, 2008. *Obesity, Fitness & Wellness Week.*

Did the gene for ADHD help our nomadic ancestors? July 5, 2008. *Obesity, Fitness & Wellness Week.*

Now you can blame those extra pounds on the ‘ice age' gene. November 22, 2008. *The Globe and Mail.*

Data on attention deficit hyperactivity disorder therapy discussed by researchers at Emory University, Department of Psychology. December 27, 2008. *Obesity, Fitness & Wellness Week.*

Research conducted at Oregon Health & Science University, Oregon National Primate Research Center has updated our knowledge about life sciences. August 16, 2008. *Obesity, Fitness & Wellness Week.*

Investigators at University of California release new data on behavior. March 29, 2008. *Obesity, Fitness & Wellness Week.*

Ol' Blue Eyes is an evolutionary mystery; Human development has taken some odd twists and turns that a number of scientists are still puzzling over. February 11, 2009. *Vancouver Sun.*

Studies in the area of attention deficit hyperactivity disorder reported from Cardiff University, Department of Psychological Medicine. February 14, 2009. *Obesity, Fitness & Wellness Week.*

Researchers at University of Nijmegen, Department of Psychiatry have published new data on attention deficit hyperactivity disorder in children. March 21, 2009. *Obesity, Fitness & Wellness Week.*

Identifying brain differences in people with ADHD. September 11, 2009. *NPR: Talk of the Nation/Science Friday.*

Studies from University of Southampton describe new findings in attention deficit hyperactivity disorder gene therapy. September 12, 2009. *Obesity, Fitness & Wellness Week.*

*2) Articles stating that the DRD4 gene is associated with ADHD but that it confers small risk*

Gene is linked to attention deficit; Discovery could lead to new drugs for hyperactivity. May 1st, 1996. *Austin American-Statesman.*

Gene abnormality traced to hyperactivity. May 1st, 1996. *Agence France-Presse.*

Genetic flaw linked to hyperactivity. May 1st, 1996. *Chicago Sun-Times.*

Gene linked to hyperactivity disorder in children. May 1st, 1996. *The Dallas Morning News.*

Scientists identify hyperactivity gene/Finding could help diagnose condition affecting estimated 2 million U.S. kids. May 1st, 1996. *Houston Chronicle.*

Gene linked to hyperactivity: UCLA Irvine finding may aid diagnosis, treating of ADHD in restless children. May 1st, 1996. *Pittsburgh Post-Gazette.*

Gene may play role in hyperactivity ADHD Hope: more drugs, less ritalin. May 1st, 1996. *The Salt Lake Tribune.*

Genetic defect linked to attention disorder // Study offers clue to how Ritalin works. May 1st, 1996. *Patriot-News.*

The possible link between genes, attention deficit. December 22, 1998. *Newsday.*

New genetic link to attention deficit hyperactivity. September 19, 2000. *The Times of India.*

The trouble with ADHD. March 18, 2001. *The Washington Post.*

Gene of the week. December 4, 2003. *The Sydney Morning Herald.*

Bad News, Good News. August 11, 2007. *Science News.*

Study findings from Semmelweis University, Institute of Medical Chemistry broaden understanding of attention deficit hyperactivity disorder in children. April 9, 2007. *Mental Health Weekly Digest.*

Study findings from Semmelweis University, Institute of Medical Chemistry broaden understanding of attention deficit hyperactivity disorder in children. April 9, 2007. *Biotech Business Week.*

Study findings from Semmelweis University, Institute of Medical Chemistry broaden understanding of attention deficit hyperactivity disorder in children. April 9, 2007. *Pharma Business Week.*

Study findings from Semmelweis University, Institute of Medical Chemistry broaden understanding of attention deficit hyperactivity disorder in children. April 10, 2007. *Life Science Weekly.*

Study findings from Semmelweis University, Institute of Medical Chemistry broaden understanding of attention deficit hyperactivity disorder in children. April 13, 2007. *Drug Week.*

Study findings from Semmelweis University, Institute of Medical Chemistry broaden understanding of attention deficit hyperactivity disorder in children. April 14, 2007. *Obesity, Fitness & Wellness Week.*

Gene predicts better outcome as cortex normalizes in teens with attention deficit hyperactivity disorder. August 6, 2007. *US Fed News.*

Gene predicts better outcome as cortex normalizes in teens with ADHD. August 6, 2007. *States News Service.*

Gene predicts better outcome as cortex normalizes in teens with ADHD. August 6, 2007. *National Institutes of Health Documents.*

Study findings from Semmelweis University, Institute of Medical Chemistry broaden understanding of attention deficit hyperactivity disorder in children. April 13, 2007. *Genomics & Genetics Weekly.*

Findings from studies by Semmelweis University, Hungary, scientists provide new insights. May 7, 2007. *Health & Medicine Week.*

Prenatal smoking increases attention-deficit/hyperactivity disorder risk in some children. April 6, 2007. *US Fed News.*

*3) Equivocal articles giving the odds ratio but concluding that the DRD4 gene is strongly associated with ADHD*

Dopamine system genes are linked to attention deficit hyperactivity disorder. August 28, 2006. *Health & Medicine Week.*

Dopamine system genes are linked to attention deficit hyperactivity disorder. August 28, 2006. *Mental Health Weekly Digest.*

Dopamine system genes are linked to attention deficit hyperactivity disorder. August 28, 2006. *Pain & Central Nervous System Week.*

Dopamine system genes are linked to attention deficit hyperactivity disorder. August 29, 2006. *Life Science Weekly.*

Dopamine system genes are linked to attention deficit hyperactivity disorder. August 30, 2006. *Biotech Week.*

Dopamine system genes are linked to attention deficit hyperactivity disorder. September 1st, 2006. *Drug Week.*

Dopamine system genes are linked to attention deficit hyperactivity disorder. September 1st, 2006. *Genomics & Genetics Weekly.*

Dopamine system genes are linked to attention deficit hyperactivity disorder. September 2, 2006. *Obesity, Fitness & Wellness Week.*

Recent Shanghai Jiao Tong University, People's Republic of China, study findings reported. December 11, 2006. *Pharma Business Week.*

Dopamine system genes are linked to attention deficit hyperactivity disorder. August 29, 2006. *Science Letter.*

Studies from National Institutes of Health yield new data on attention deficit hyperactivity disorder therapy. May 21, 2007. *Biotech Business Week.*

Studies from National Institutes of Health yield new data on attention deficit hyperactivity disorder therapy. May 21, 2007. *Mental Health Weekly Digest.*

Studies from National Institutes of Health yield new data on attention deficit hyperactivity disorder therapy. May 21, 2007. *Health & Medicine Week.*

Studies from National Institutes of Health yield new data on attention deficit hyperactivity disorder therapy. May 21, 2007. *Pharma Business Week.*

Studies from National Institutes of Health yield new data on attention deficit hyperactivity disorder therapy. May 22, 2007. *Life Science Weekly.*

Studies from National Institutes of Health yield new data on attention deficit hyperactivity disorder therapy. May 22, 2007. *Science Letter.*

Studies from National Institutes of Health yield new data on attention deficit hyperactivity disorder therapy. May 25, 2007. *Drug Week.*

Studies from National Institutes of Health yield new data on attention deficit hyperactivity disorder therapy. May 26, 2007. *Obesity, Fitness & Wellness Week.*

Researchers' findings from Shanghai Jiao Tong University, People's Republic of China, advance research. January 15, 2007. *Biotech Business Week.*

People's Republic of China; Shanghai Jiao Tong University, People's Republic of China, researchers publish recent findings. May 1st, 2007. *Science Letter.*

Shanghai Jiao Tong University, People's Republic of China, study data released. June 11, 2007. *Health & Medicine Week.*

Researchers' work from Shanghai Jiao Tong University, People's Republic of China, adds to body of knowledge. July 3, 2007. *Science Letter.*

Research from Canada, the People's Republic of China and Germany in attention deficit hyperactivity disorders provides new insights. July 31, 2007. *Science Letter.*

Studies from Shanghai Jiao Tong University, People's Republic of China, highlight latest findings. August 14, 2007. *Life Science Weekly.*

Researchers in Canada, Germany and the People's Republic of China publish new attention deficit hyperactivity disorders data. August 27, 2007. *Health & Medicine Week.*

Journal articles present study results from Shanghai Jiao Tong University, People's Republic of China. September 4, 2007. *Life Science Weekly.*

*4) Articles stating that the DRD4 gene is not significantly associated with ADHD*

Studies from University of Hong Kong in the area of attention deficit hyperactivity disorder in children published. January 1st, 2007. *Mental Health Weekly Digest*

Investigators at Semmelweis University release new data on attention deficit hyperactivity disorder. February 7, 2009. *Obesity, Fitness & Wellness Week*
