# Supplementary material for: Misrepresentation of Neuroscience Data Might Give Rise to Misleading Conclusions in the Media: The Case of Attention Deficit Hyperactivity Disorder
Source: PLoS One. 2011 Jan 31;6(1):e14618. doi: 10.1371/journal.pone.0014618 (PMC3031509; doi:10.1371/journal.pone.0014618)
Supplement: Text S3 — Scientific studies performed in mice and related to ADHD and media articles that echoed to the 3 scientific articles given in Table 3. (0.08 MB DOC) [file pone.0014618.s004.doc]

**Supporting Text S3**

**1) Scientific studies performed in mouse brain and related to ADHD (101 articles)**

*1.1) Forty five articles without overstatement.*

Akahoshi, E, Yoshimura, S, Uruno, S, Ishihara-Sugano, M (2009) Effect of dioxins on regulation of tyrosine hydroxylase gene expression by aryl hydrocarbon receptor: a neurotoxicology study. *Environ Health* **8**: 24.

Balcioglu A, Ren JQ, McCarthy D, Spencer TJ, Biederman J, Bhide PG (2009) Plasma and brain concentrations of oral therapeutic doses of methylphenidate and their impact on brain monoamine content in mice. Neuropharmacology 57:687-693.

Belke, TW, Garland, T, Jr. (2007) A brief opportunity to run does not function as a reinforcer for mice selected for high daily wheel-running rates. *J Exp Anal Behav* **88**: 199-213.

Davies, W, Humby, T, Isles, AR, Burgoyne, PS, Wilkinson, LS (2007) X-monosomy effects on visuospatial attention in mice: a candidate gene and implications for Turner syndrome and attention deficit hyperactivity disorder. *Biol Psychiatry* **61**: 1351-1360.

Davis JA, Gould TJ (2007) Atomoxetine reverses nicotine withdrawal-associated deficits in contextual fear conditioning. *Neuropsychopharmacology* 32:2011-2019.

Ding, YS *et al* (2004) Brain kinetics of methylphenidate (Ritalin) enantiomers after oral administration. *Synapse* **53**: 168-175.

Ehrlich ME, Sommer J, Canas E, Unterwald EM (2002) Periadolescent mice show enhanced DeltaFosB upregulation in response to cocaine and amphetamine. J Neurosci 22:9155-9159.

Fasano C, Poirier A, DesGroseillers L, Trudeau LE (2008) Chronic activation of the D2 dopamine autoreceptor inhibits synaptogenesis in mesencephalic dopaminergic neurons in vitro. Eur J Neurosci 28:1480-1490.

Fukui, R *et al* (2003) Effect of methylphenidate on dopamine/DARPP signalling in adult, but not young, mice. *J Neurochem* **87**: 1391-1401.

Galinanes, GL, Taravini, IR, Murer, MG (2009) Dopamine-dependent periadolescent maturation of corticostriatal functional connectivity in mouse. *J Neurosci* **29**: 2496-2509.

Gould TJ, Rukstalis M, Lewis MC (2005) Atomoxetine and nicotine enhance prepulse inhibition of acoustic startle in C57BL/6 mice. Neurosci Lett 377:85-90.

Guerriero, RM, Hayes, MM, Dhaliwal, SK, Ren, JQ, Kosofsky, BE (2006) Preadolescent methylphenidate versus cocaine treatment differ in the expression of cocaine-induced locomotor sensitization during adolescence and adulthood. *Biol Psychiatry* **60**: 1171-1180.

Hawken, CM, Brown, RE, Carrey, N, Wilkinson, M (2004) Long-term methylphenidate treatment down-regulates c-fos in the striatum of male CD-1 mice. *Neuroreport* **15**: 1045-1048.

Helms, CM, Gubner, NR, Wilhelm, CJ, Mitchell, SH, Grandy, DK (2008) D4 receptor deficiency in mice has limited effects on impulsivity and novelty seeking. *Pharmacol Biochem Behav* **90**: 387-393.

Hu, YF, Caron, MG, Sieber-Blum, M (2009) Norepinephrine transport-mediated gene expression in noradrenergic neurogenesis. *BMC Genomics* **10**: 151.

Hu, Z, Cooper, M, Crockett, DP, Zhou, R (2004) Differentiation of the midbrain dopaminergic pathways during mouse development. *J Comp Neurol* **476**: 301-311.

Isles, AR, Humby, T, Walters, E, Wilkinson, LS (2004) Common genetic effects on variation in impulsivity and activity in mice. *J Neurosci* **24**: 6733-6740.

Issy AC, Salum C, Del Bel EA (2009) Nitric oxide modulation of methylphenidate-induced disruption of prepulse inhibition in Swiss mice. Behav Brain Res 205:475-481.

Itzhak, Y, Martin, JL (2002) Effect of the neuronal nitric oxide synthase inhibitor 7-nitroindazole on methylphenidate-induced hyperlocomotion in mice. *Behav Pharmacol* **13**: 81-86.

Jones, LG *et al* (2008) Lead exposure during development results in increased neurofilament phosphorylation, neuritic beading, and temporal processing deficits within the murine auditory brainstem. *J Comp Neurol* **506**: 1003-1017.

Kharkar, PS *et al* (2009) Synthesis and biological characterization of (3R,4R)-4-(2-(benzhydryloxy)ethyl)-1-((R)-2-hydroxy-2-phenylethyl)-piperidin-3-ol and its stereoisomers for activity toward monoamine transporters. *ChemMedChem* **4**: 1075-1085.

Kim, Y *et al* (2009) Methylphenidate-induced dendritic spine formation and DeltaFosB expression in nucleus accumbens. *Proc Natl Acad Sci U S A* **106**: 2915-2920.

Kimura-Kuroda, J, Nagata, I, Kuroda, Y (2007) Disrupting effects of hydroxy-polychlorinated biphenyl (PCB) congeners on neuronal development of cerebellar Purkinje cells: a possible causal factor for developmental brain disorders? *Chemosphere* **67**: S412-420.

King, SL *et al* (2003) Conditional expression in corticothalamic efferents reveals a developmental role for nicotinic acetylcholine receptors in modulation of passive avoidance behavior. *J Neurosci* **23**: 3837-3843.

Koike, H *et al* (2009) Behavioral abnormality and pharmacologic response in social isolation-reared mice. *Behav Brain Res* **202**: 114-121.

Koot, S, van den Bos, R, Adriani, W, Laviola, G (2009) Gender differences in delay-discounting under mild food restriction. *Behav Brain Res* **200**: 134-143.

Lau, K, McLean, WG, Williams, DP, Howard, CV (2006) Synergistic interactions between commonly used food additives in a developmental neurotoxicity test. *Toxicol Sci* **90**: 178-187.

Lu, R *et al* (2009) Epitope-tagged receptor knock-in mice reveal that differential desensitization of alpha2-adrenergic responses is because of ligand-selective internalization. *J Biol Chem* **284**: 13233-13243.

McFadyen, MP, Brown, RE, Carrey, N (2002) Subchronic methylphenidate administration has no effect on locomotion, emotional behavior, or water maze learning in prepubertal mice. *Dev Psychobiol* **41**: 123-132.

Montgomery, KS *et al* (2008) Chronic, low-dose prenatal exposure to methylmercury impairs motor and mnemonic function in adult C57/B6 mice. *Behav Brain Res* **191**: 55-61.

Moon J, Beaudin AE, Verosky S, Driscoll LL, Weiskopf M, Levitsky DA, Crnic LS, Strupp BJ (2006) Attentional dysfunction, impulsivity, and resistance to change in a mouse model of fragile X syndrome. Behav Neurosci 120:1367-1379.

Patel, S, Stolerman, IP, Asherson, P, Sluyter, F (2006) Attentional performance of C57BL/6 and DBA/2 mice in the 5-choice serial reaction time task. *Behav Brain Res* **170**: 197-203.

Paz, R, Barsness, B, Martenson, T, Tanner, D, Allan, AM (2007) Behavioral teratogenicity induced by nonforced maternal nicotine consumption. *Neuropsychopharmacology* **32**: 693-699.

Penner, MR *et al* (2002) Age-related distribution of c-fos expression in the striatum of CD-1 mice after acute methylphenidate administration. *Brain Res Dev Brain Res* **135**: 71-77.

Powell, SB, Paulus, MP, Hartman, DS, Godel, T, Geyer, MA (2003) RO-10-5824 is a selective dopamine D4 receptor agonist that increases novel object exploration in C57 mice. *Neuropharmacology* **44**: 473-481.

Rondou, P, Haegeman, G, Vanhoenacker, P, Van Craenenbroeck, K (2008) BTB Protein KLHL12 targets the dopamine D4 receptor for ubiquitination by a Cul3-based E3 ligase. *J Biol Chem* **283**: 11083-11096.

Schaefer TL, Vorhees CV, Williams MT (2009) Mouse plasmacytoma-expressed transcript 1 knock out induced 5-HT disruption results in a lack of cognitive deficits and an anxiety phenotype complicated by hypoactivity and defensiveness. *Neuroscience* 164:1431-1443.

Suter, W, Martus, HJ, Elhajouji, A (2006) Methylphenidate is not clastogenic in cultured human lymphocytes and in the mouse bone-marrow micronucleus test. *Mutat Res* **607**: 153-159.

Tanida, T *et al* (2009) Fetal and neonatal exposure to three typical environmental chemicals with different mechanisms of action: mixed exposure to phenol, phthalate, and dioxin cancels the effects of sole exposure on mouse midbrain dopaminergic nuclei. *Toxicol Lett* **189**: 40-47.

Teo, SK *et al* (2003) D-Methylphenidate is non-genotoxic in in vitro and in vivo assays. *Mutat Res* **537**: 67-79.

Tilley, MR, Gu, HH (2008) The effects of methylphenidate on knockin mice with a methylphenidate-resistant dopamine transporter. *J Pharmacol Exp Ther* **327**: 554-560.

Van Craenenbroeck, K *et al* (2006) Influence of the antipsychotic drug pipamperone on the expression of the dopamine D4 receptor. *Life Sci* **80**: 74-81.

Wood, SC, Anagnostaras, SG (2009) Memory and psychostimulants: modulation of Pavlovian fear conditioning by amphetamine in C57BL/6 mice. *Psychopharmacology (Berl)* **202**: 197-206.

Zhu, HJ *et al* (2006) The role of the polymorphic efflux transporter P-glycoprotein on the brain accumulation of d-methylphenidate and d-amphetamine. *Drug Metab Dispos* **34**: 1116-1121.

1.2) Thirty three articles with overstatements of type 1.

Avale ME, Falzone TL, Gelman DM, Low MJ, Grandy DK, Rubinstein M (2004) The dopamine D4 receptor is essential for hyperactivity and impaired behavioral inhibition in a mouse model of attention deficit/hyperactivity disorder. *Mol Psychiatry* 9:718-726.

Bruno KJ, Freet CS, Twining RC, Egami K, Grigson PS, Hess EJ (2007) Abnormal latent inhibition and impulsivity in coloboma mice, a model of ADHD. *Neurobiol Dis* 25:206-216.

Cowen MS, Schroff KC, Gass P, Sprengel R, Spanagel R (2003) Neurobehavioral effects of alcohol in AMPA receptor subunit (GluR1) deficient mice. *Neuropharmacology* 45:325-333.

Davies W, Humby T, Kong W, Otter T, Burgoyne PS, Wilkinson LS (2009) Converging pharmacological and genetic evidence indicates a role for steroid sulfatase in attention. *Biol Psychiatry* 66:360-367.

Fredriksson A, Archer T (2003) Hyperactivity following postnatal NMDA antagonist treatment: reversal by D-amphetamine. *Neurotox Res* 5:549-564.

Fredriksson A, Archer T (2004) Neurobehavioural deficits associated with apoptotic neurodegeneration and vulnerability for ADHD. *Neurotox Res* 6:435-456.

Granon S, Faure P, Changeux JP (2003) Executive and social behaviors under nicotinic receptor regulation. *Proc Natl Acad Sci U S A* 100:9596-9601.

Jones MD, Hess EJ (2003) Norepinephrine regulates locomotor hyperactivity in the mouse mutant coloboma. *Pharmacol Biochem Behav* 75:209-216.

Jones MD, Williams ME, Hess EJ (2001) Abnormal presynaptic catecholamine regulation in a hyperactive SNAP-25-deficient mouse mutant. *Pharmacol Biochem Behav* 68:669-676.

Kajiwara Y, Buxbaum JD, Grice DE (2009) SLITRK1 binds 14-3-3 and regulates neurite outgrowth in a phosphorylation-dependent manner. *Biol Psychiatry* 66:918-925.

Lafenetre P, Chaouloff F, Marsicano G (2009) Bidirectional regulation of novelty-induced behavioral inhibition by the endocannabinoid system. *Neuropharmacology* 57:715-721.

Lahdesmaki J, Sallinen J, MacDonald E, Scheinin M (2004) Alpha2A-adrenoceptors are important modulators of the effects of D-amphetamine on startle reactivity and brain monoamines. *Neuropsychopharmacology* 29:1282-1293.

McDonald MP, Wong R, Goldstein G, Weintraub B, Cheng SY, Crawley JN (1998) Hyperactivity and learning deficits in transgenic mice bearing a human mutant thyroid hormone beta1 receptor gene. *Learn Mem* 5:289-301.

Moore TM, Brown T, Cade M, Eells JB (2008) Alterations in amphetamine-stimulated dopamine overflow due to the Nurr1-null heterozygous genotype and postweaning isolation. *Synapse* 62:764-774.

Noain D, Avale ME, Wedemeyer C, Calvo D, Peper M, Rubinstein M (2006) Identification of brain neurons expressing the dopamine D4 receptor gene using BAC transgenic mice. *Eur J Neurosci* 24:2429-2438.

Pascoli V, Valjent E, Corbille AG, Corvol JC, Tassin JP, Girault JA, Herve D (2005) cAMP and extracellular signal-regulated kinase signaling in response to d-amphetamine and methylphenidate in the prefrontal cortex in vivo: role of beta 1-adrenoceptors. *Mol Pharmacol* 68:421-429.

Qu S, Le W, Zhang X, Xie W, Zhang A, Ondo WG (2007) Locomotion is increased in a11-lesioned mice with iron deprivation: a possible animal model for restless legs syndrome. *J Neuropathol Exp Neurol* 66:383-388.

Raber J, Mehta PP, Kreifeldt M, Parsons LH, Weiss F, Bloom FE, Wilson MC (1997) Coloboma hyperactive mutant mice exhibit regional and transmitter-specific deficits in neurotransmission. *J Neurochem* 68:176-186.

Rhodes JS, Garland T (2003) Differential sensitivity to acute administration of Ritalin, apomorphine, SCH 23390, but not raclopride in mice selectively bred for hyperactive wheel-running behavior. *Psychopharmacology* (Berl) 167:242-250.

Rhodes JS, Hosack GR, Girard I, Kelley AE, Mitchell GS, Garland T, Jr. (2001) Differential sensitivity to acute administration of cocaine, GBR 12909, and fluoxetine in mice selectively bred for hyperactive wheel-running behavior. *Psychopharmacology* (Berl) 158:120-131.

Ruocco LA, Viggiano D, Pignatelli M, Iannaccone T, Rimoli MG, Melisi D, Curcio A, De Lucia S, Carboni E, Gironi Carnevale UA, de Caprariis P, Sadile AG (2008) Galactosilated dopamine increases attention without reducing activity in C57BL/6 mice. *Behav Brain Res* 187:449-454.

Sackler AM, Weltman AS (1985) Effects of methylphenidate on whirler mice: an animal model for hyperkinesis. *Life Sci* 37:425-431.

Siesser WB, Cheng SY, McDonald MP (2005) Hyperactivity, impaired learning on a vigilance task, and a differential response to methylphenidate in the TRbetaPV knock-in mouse. *Psychopharmacology (Berl)* 181:653-663.

Siesser WB, Zhao J, Miller LR, Cheng SY, McDonald MP (2006) Transgenic mice expressing a human mutant beta1 thyroid receptor are hyperactive, impulsive, and inattentive. *Genes Brain Behav* 5:282-297.

Steffensen SC, Wilson MC, Henriksen SJ (1996) Coloboma contiguous gene deletion encompassing Snap alters hippocampal plasticity. *Synapse* 22:281-289.

Stein JM, Bergman W, Fang Y, Davison L, Brensinger C, Robinson MB, Hecht NB, Abel T (2006) Behavioral and neurochemical alterations in mice lacking the RNA-binding protein translin. *J Neurosci* 26:2184-2196.

Tanaka K, Shintani N, Hashimoto H, Kawagishi N, Ago Y, Matsuda T, Hashimoto R, Kunugi H, Yamamoto A, Kawaguchi C, Shimada T, Baba A (2006) Psychostimulant-induced attenuation of hyperactivity and prepulse inhibition deficits in Adcyap1-deficient mice. *J Neurosci* 26:5091-5097.

Thomas TC, Grandy DK, Gerhardt GA, Glaser PE (2009) Decreased dopamine D4 receptor expression increases extracellular glutamate and alters its regulation in mouse striatum. *Neuropsychopharmacology* 34:436-445.

Trantham-Davidson H, Vazdarjanova A, Dai R, Terry A, Bergson C (2008) Up-regulation of calcyon results in locomotor hyperactivity and reduced anxiety in mice. *Behav Brain Res* 189:244-249.

Trinh JV, Nehrenberg DL, Jacobsen JP, Caron MG, Wetsel WC (2003) Differential psychostimulant-induced activation of neural circuits in dopamine transporter knockout and wild type mice. *Neuroscience* 118:297-310.

Tsuchida R, Kubo M, Shintani N, Abe M, Koves K, Uetsuki K, Kuroda M, Hashimoto H, Baba A (2009) Inhibitory effects of osemozotan, a serotonin 1A-receptor agonist, on methamphetamine-induced c-Fos expression in prefrontal cortical neurons. *Biol Pharm Bull* 32:728-731.

Vincent SG, Waddell AE, Caron MG, Walker JK, Fisher JT (2007) A murine model of hyperdopaminergic state displays altered respiratory control. *Faseb J* 21:1463-1471.

Yuen EY, Yan Z (2009) Dopamine D4 receptors regulate AMPA receptor trafficking and glutamatergic transmission in GABAergic interneurons of prefrontal cortex. *J Neurosci* 29:550-562.

*1.3) Twenty three articles that reinforced the type 1 overstatement with a claim about its clinical relevance.*

Barr, AM *et al* (2004) The selective serotonin-2A receptor antagonist M100907 reverses behavioral deficits in dopamine transporter knockout mice. *Neuropsychopharmacology* **29**: 221-228.

Beaulieu, JM, Sotnikova, TD, Gainetdinov, RR, Caron, MG (2006) Paradoxical striatal cellular signaling responses to psychostimulants in hyperactive mice. *J Biol Chem* **281**: 32072-32080.

Beaulieu, JM *et al* (2004) Lithium antagonizes dopamine-dependent behaviors mediated by an AKT/glycogen synthase kinase 3 signaling cascade. *Proc Natl Acad Sci U S A* **101**: 5099-5104.

Besson, M, Suarez, S, Cormier, A, Changeux, JP, Granon, S (2008) Chronic nicotine exposure has dissociable behavioural effects on control and beta2-/- mice. *Behav Genet* **38**: 503-514.

Bruno, KJ, Hess, EJ (2006) The alpha(2C)-adrenergic receptor mediates hyperactivity of coloboma mice, a model of attention deficit hyperactivity disorder. *Neurobiol Dis* **23**: 679-688.

Fan, X, Hess, EJ (2007) D2-like dopamine receptors mediate the response to amphetamine in a mouse model of ADHD. *Neurobiol Dis* **26**: 201-211.

Fetsko, LA, Xu, R, Wang, Y (2003) Alterations in D1/D2 synergism may account for enhanced stereotypy and reduced climbing in mice lacking dopamine D2L receptor. *Brain Res* **967**: 191-200.

Fox, GB *et al* (2005) Pharmacological properties of ABT-239 [4-(2-{2-[(2R)-2-Methylpyrrolidinyl]ethyl}-benzofuran-5-yl)benzonitrile]: II. Neurophysiological characterization and broad preclinical efficacy in cognition and schizophrenia of a potent and selective histamine H3 receptor antagonist. *J Pharmacol Exp Ther* **313**: 176-190.

Franowicz, JS *et al* (2002) Mutation of the alpha2A-adrenoceptor impairs working memory performance and annuls cognitive enhancement by guanfacine. *J Neurosci* **22**: 8771-8777.

Gainetdinov, RR *et al* (1999) Role of serotonin in the paradoxical calming effect of psychostimulants on hyperactivity. *Science* **283**: 397-401.

Lalonde R, Strazielle C (2009) The relation between open-field and emergence tests in a hyperactive mouse model. *Neuropharmacology* 57:722-724.

Lammel, S *et al* (2008) Unique properties of mesoprefrontal neurons within a dual mesocorticolimbic dopamine system. *Neuron* **57**: 760-773.

Olmstead, MC, Ouagazzal, AM, Kieffer, BL (2009) Mu and delta opioid receptors oppositely regulate motor impulsivity in the signaled nose poke task. *PLoS One* **4**: e4410.

Smith, DG *et al* (2008) Behavioral and biochemical responses to d-amphetamine in MCH1 receptor knockout mice. *Synapse* **62**: 128-136.

Tan, CM, Wilson, MH, MacMillan, LB, Kobilka, BK, Limbird, LE (2002) Heterozygous alpha 2A-adrenergic receptor mice unveil unique therapeutic benefits of partial agonists. *Proc Natl Acad Sci U S A* **99**: 12471-12476.

Tzavara, ET *et al* (2004) M4 muscarinic receptors regulate the dynamics of cholinergic and dopaminergic neurotransmission: relevance to the pathophysiology and treatment of related CNS pathologies. *Faseb J* **18**: 1410-1412.

Tzavara, ET *et al* (2006) Endocannabinoids activate transient receptor potential vanilloid 1 receptors to reduce hyperdopaminergia-related hyperactivity: therapeutic implications. *Biol Psychiatry* **59**: 508-515.

Vukhac, KL, Sankoorikal, EB, Wang, Y (2001) Dopamine D2L receptor- and age-related reduction in offensive aggression. *Neuroreport* **12**: 1035-1038.

Weiss, S, Nosten-Bertrand, M, McIntosh, JM, Giros, B, Martres, MP (2007a) Nicotine improves cognitive deficits of dopamine transporter knockout mice without long-term tolerance. *Neuropsychopharmacology* **32**: 2465-2478.

Weiss, S *et al* (2007b) Functional alterations of nicotinic neurotransmission in dopamine transporter knock-out mice. *Neuropharmacology* **52**: 1496-1508.

Wu, N, Cepeda, C, Zhuang, X, Levine, MS (2007) Altered corticostriatal neurotransmission and modulation in dopamine transporter knock-down mice. *J Neurophysiol* **98**: 423-432.

Xiu, X, Puskar, NL, Shanata, JA, Lester, HA, Dougherty, DA (2009) Nicotine binding to brain receptors requires a strong cation-pi interaction. *Nature* **458**: 534-537.

Zhuang, X *et al* (2001) Hyperactivity and impaired response habituation in hyperdopaminergic mice. *Proc Natl Acad Sci U S A* **98**: 1982-1987.

**2) Media articles that echoed to the 3 scientific articles given in Table 3**

*2.1) Media articles echoing to Dougherty et al (1999)*

*2.1.1) Without further comment*

Brain scan find may help children --- Could detect attention deficit disorder: Study. December 17, 1999. *The Toronto Star. Toronto, Canada*

New brain scan could help diagnose attention deficit disorder. December 17, 1999. *Associated Press Newswires. USA*

National association hopeful about medical test to diagnose AD/HD. December 17, 1999. *Business Wire. USA*

The Lancet Publishes Clinical Results of Boston Life Sciences' Altropane for Use in the Diagnosis of Attention Deficit Hyperactivity Disorder. December 17, 1999. *Business Wire. USA*

Breakthrough claimed for attention disorders. December 17, 1999. *The Commercial Appeal Memphis. Memphis, TN*

Brain scans reveal ADHD Study shows biochemical difference in attention deficit disorder. December 17, 1999. *Denver Post. Denver, CO*

Boston Life: Altropane Study 'Significant'. December 17, 1999. *Dow Jones News Service. USA*

Brain scan may help diagnose attention disorder. December 17, 1999. *The Florida Times-Union. Jacksonville, Florida*

New brain scan could help diagnose ADHD. December 17, 1999. *The Grand Rapids Press. Grand Rapids MI*

Brain scan may detect ADHD. December 17, 1999. *Greensboro News & Record. Greensboro, NC*

Brain scans link ADHD to chemical imbalance: the finding points toward a way to reduce the number of children misdiagnosed with the behavioral malady. December 17, 1999. *The Oregonian. Portland, Oregon*

Nation/World: Brain scans help detect ADHD. December 17, 1999. *Patriot-News. Harrisburg, Pennsylvania*

World: Chemical differences found for ADHD. December 17, 1999. *The Plain Dealer. Cleveland*

Research notes - ADHD: brain scans find biochemical abnormalities. December 17, 1999. *American Health Line. USA*

Discovery could help diagnose ADHD. December 17, 1999. *The Salt Lake Tribune. Salt Lake City, Utah*

Attention deficit id'd in brain chemistry the study is first to show abnormality in sufferers. December 17, 1999. *Seattle Post-Intelligencer. Seattle*

Science briefs: Study hints at test for attention deficit. December 17, 1999. *Times-Picayune. New Orleans*

Brain scans reveal ADHD. December 17, 1999. *The Topeka Capital-Journal. Topeka, Kansas*

A dose of praise for Ritalin. December 21, 1999. *The Globe and Mail. Canada*

Scans may pin down ADHD. December 17, 1999. *Tulsa World. Tulsa, Oklahoma*

New test could help diagnose attention deficit disorder. December 18, 1999. *The Cambridge Reporter. Cambridge, Canada*

New brain scan may help diagnose attention deficit. December 18, 1999. *Houston Chronicle. Houston, Texas*

Focus on: Health, worth our attention. December 19, 1999. *The Boston Globe. Boston*

Boston Life Sciences Advances Start of Clinical Trial for Attention Deficit Hyperactivity Disorder; Clinical Development Based On Recently-Reported Results Published in The Lancet. January 25, 2000. *Business Wire. USA*

Scans show brain difference in those with ADHD. December 17, 1999. *Reuters News. UK*

Shedding light on hyperactivity. February 6, 2000. *The Boston Globe. The Boston Globe*

*2.1.2) With addition of a mitigating comment*

*New brain scan may diagnose ADHD. December 16, 1999. Associated Press Online. USA.*

New brain scan links biochemical abnormalities to ADHD. December 17, 1999. *The Baton Rouge Advocate. Baton Rouge, LA*

Attention-getting research - Brain scans link biochemical differences and hyperactivity disorder. December 17, 1999. *The Star-Ledger Newark. Newark, NJ*

Scan could become part of evaluating hyperactivity. December 17, 1999. *Pittsburgh Post-Gazette. Pittsburgh*

Scan may spot attention deficit: Sufferers are found to have distinct chemical patterns in the brain. December 17, 1999. *The Orange County Register. Orange CA*

Chemical imbalance tied to Attention Disorder. December 17, 1999. *Omaha World-Herald. Omaha, Nebraska*

Brain scans seen as test in attention disorder. December 17, 1999. *The Boston Globe. Boston*

Scan could spot attention disorder. December 18, 1999. *Deseret News. Salt Lake City, Utah*

New brain scan could help diagnose attention deficit disorder. December 19, 1999. *Associated Press Newswires. USA*

*2.2) Media articles echoing to Gainetdinov et al (1999)*

*2.2.1) Without further comment*

Hyperactivity drug researched. January 14, 1999. *Associated Press Online. USA*

Researchers says ADHD drug ritalin works by affecting serotonin. January 15, 1999. *Dow Jones Business News. USA*

Researchers find out how drug calms hyper kids. January 15, 1999. *Deseret News. Deseret, Utah*

Researchers find how drug helps calm hyperactive kids. January 15, 1999. *The Grand Rapids Press. Grand Rapids*

Study pinpoints how drugs calm hyperactivity. January 15, 1999. *The Patriot Ledger Quincy. Quincy, MA*

Study finds how drugs calm hyperactivity in kids. January 15, 1999. *The Record. Hackensack, NJ*

Researchers find how drug calms hyperactive children. January 15, 1999. *Associated Press Newswires. USA*

Study on mice finds clues on brain drugs. January 15, 1999. *Times Union. Albany NY*

Duke U. scientists discover role for serotonin in ADHD. January 18, 1999. *U-WIRE. USA*

Explaining the Ritalin paradox. January 25, 1999. *The Boston Globe. Boston*

Briefing. January 28, 1999. *Times Union. Albany,N.Y.*

Healthbriefing. January 29, 1999. *Times-Picayune. New Orleans*

New light shed on Ritalin's effect. January 15, 1999. *Chicago Sun-Times. Chicago*

Study shifts thought on how Ritalin works. January 15, 1999. *The Orange County Register. Orange, CA*

Study suggests doses of stimulants useful in controlling hyperactivity. January 15, 1999. *The Plain Dealer. Cleveland*

Hyper mice reveal ritalin's main mode of action: serotonin, not dopamine alone, calm kids's ADHD, Duke scientists report. January 19, 1999. *BIOWORLD Today. USA*

Brain on drugs: Hyperactive ingredients. February 1, 1999. *York Daily Record. York P.A.*

Family briefs: Sources of hype. February 7, 1999. *Austin American-Statesman. Austin, Texas*

*2.2.2) With addition of a mitigating comment*

Study points to better children's disorder drugs. January 14, 1999. *Reuters News. UK*

Findings: Better Attention Deficit drugs possible. January 15, 1999. *The Washington Post. Washington DC*

*2.3) Media articles echoing to Cardinal et al (1999) without further comment*

Impulsive? It's all in the brain. May 25, 2001. *The Express. London, UK*

Impulsive behaviour is linked to brain damage. May 25, 2001. *The Herald. Glasgow, UK*

Acting on impulse may be all in your head. May 24, 2001. *Evening News - Scotland. Edinburgh, UK*

Pleasing find on gratification. May 25, 2001. *Financial Times. London, UK*

New insight on impulsive behaviour. May 25, 2001. *The Daily Telegraph. London, UK*

New study pinpoints source of impulsive behavior. May 24, 2001. *Reuters News. UK*

Unlocking impulsiveness. June 5, 2001. *Illawarra Mercury. Wollongong, Australia*

Identity crisis not mind over matter. June 2, 2001. *Gold Coast Bulletin. Queensland, Australia*
